# Living Kidney Donation: A Narrative Review of Mid- and Long-term Psychosocial Outcomes

**DOI:** 10.1097/TP.0000000000005094

**Published:** 2024-06-18

**Authors:** Emma K. Massey, Andrew D. Rule, Arthur J. Matas

**Affiliations:** 1 Erasmus Medical Center Transplant Institute, University Medical Center Rotterdam, Department of Internal Medicine, Rotterdam, Zuid Holland, the Netherlands.; 2 Division of Nephrology and Hypertension, Mayo Clinic, Rochester, MN.; 3 Department of Surgery, Transplantation Division, University of Minnesota, Minneapolis, MN.

## Abstract

**Abstract.** Living kidney donors make a significant contribution to alleviating the organ shortage. The aim of this article is to provide an overview of mid- and long-term (≥12 mo) living donor psychosocial outcomes and highlight areas that have been understudied and should be immediately addressed in both research and clinical practice. We conducted a narrative review by searching 3 databases. A total of 206 articles were included. Living donors can be divided into those who donate to an emotionally or genetically related person, the so-called directed donors, or to an emotionally or genetically unrelated recipient, the so-called nondirected donors. The most commonly investigated (bio)psychosocial outcome after living donation was health-related quality of life. Other generic (bio)psychological outcomes include specific aspects of mental health such as depression, and fatigue and pain. Social outcomes include financial and employment burdens and problems with insurance. Donation-specific psychosocial outcomes include regret, satisfaction, feelings of abandonment and unmet needs, and benefits of living kidney donation. The experience of living donation is complex and multifaceted, reflected in the co-occurrence of both benefits and burden after donation. Noticeably, no interventions have been developed to improve mid- or long-term psychosocial outcomes among living donors. We highlight areas for methodological improvement and identified 3 areas requiring immediate attention from the transplant community in both research and clinical care: (1) recognizing and providing care for the minority of donors who have poorer long-term psychosocial outcomes after donation, (2) minimizing donation-related financial burden, and (3) studying interventions to minimize long-term psychosocial problems.

## INTRODUCTION

The consequences of receiving a kidney from a living donor (LD) for individuals with end-stage kidney failure are considerable: avoiding lengthy waiting time on the waiting list, receiving an organ from a screened and healthy donor, a planned surgery, and better survival chances when compared with alternative treatments and higher quality of life (QoL).^1-3^ Given these advantages for both the recipient and society, we have a duty of care to provide LDs with optimal education, support, and treatment, which requires understanding the impact of donation on health outcomes. To fully understand the impact of living donation on health outcomes, it is important to assess biological, psychological, and social domains^4,5^ because they each contribute both individually and interact to influence health. Much research has focused on medical and surgical aspects of living kidney donation.^6,7^ In recent years, there has been greater attention to the psychosocial impact, particularly within the first 12 mo.^3^ To minimize risks for LDs, it is important to have a thorough understanding of the mid- and long-term psychosocial outcomes and factors associated with poorer outcomes. This knowledge is essential to inform clinical practice and policy on education and screening of potential donor candidates. Because living donation programs and practices continually develop over time, it is essential that this knowledge is up-to-date. Therefore, we aim to provide an overview of the findings on mid- and long-term generic and donation-specific psychosocial outcomes and highlight areas that have been understudied and should be immediately addressed in both research and clinical practice. When possible, we differentiate findings according to the type of donor.

### Terminology in Living Kidney Donation

Various terms are used to refer to the same groups of individuals. In America, donors are generally referred to as direct or nondirected donors (NDDs). A “directed” donor is an individual who donates a kidney to a designated recipient with whom they have a genetic or emotional relationship. A “nondirected” kidney donor is an individual who wishes to donate a kidney but does not have a designated recipient and is willing to donate to any candidate on the waiting list, usually in an anonymous procedure.^8^ In Europe, NDDs have been referred to as “unspecified” donors.^9^ Other terms for NDDs include “anonymous,” “altruistic,” or “Good Samaritan” donors. Terms such as “altruistic” can be applied to donors in all LDs as they refer to the motivation or principles of the donor and are therefore less useful for a specific group. A final category, referred to as social media donors, or “directed altruistic donors,” is individuals who respond to a (social) media campaign and subsequently donate to a specific individual with whom they have no emotional or genetic relationship. They may or may not have met the recipient; however, they are not anonymous to them.

## METHODOLOGY

A narrative literature review was conducted to provide a comprehensive overview of the literature on mid- and long-term psychosocial outcomes after living kidney donation. Databases Medline (all) and Embase were searched from inception until August 2023. This resulted in 1588 articles being found after the removal of duplicates. Only English-language articles using human participants and those focusing on living kidney donors were included. As the focus was on mid- and long-term outcomes, only articles including data collection at or after 12 mo postdonation were included. For retrospective articles, an average of ≥12 mo time since donation was used as the inclusion criterion. Articles focusing exclusively on recipients, the general population, predonation, or medical/physical/surgical outcomes among donors were excluded. Conference abstracts, single-case studies, personal viewpoints, study protocols, commentaries, and editorials were excluded. Articles focusing exclusively on individuals who were denied for donation were also excluded. Two relevant articles known to the authors not captured in the search were added. See **Appendix A** (**SDC,** http://links.lww.com/TP/D77) for the search string.

## RESULTS

**FIGURE 1.**
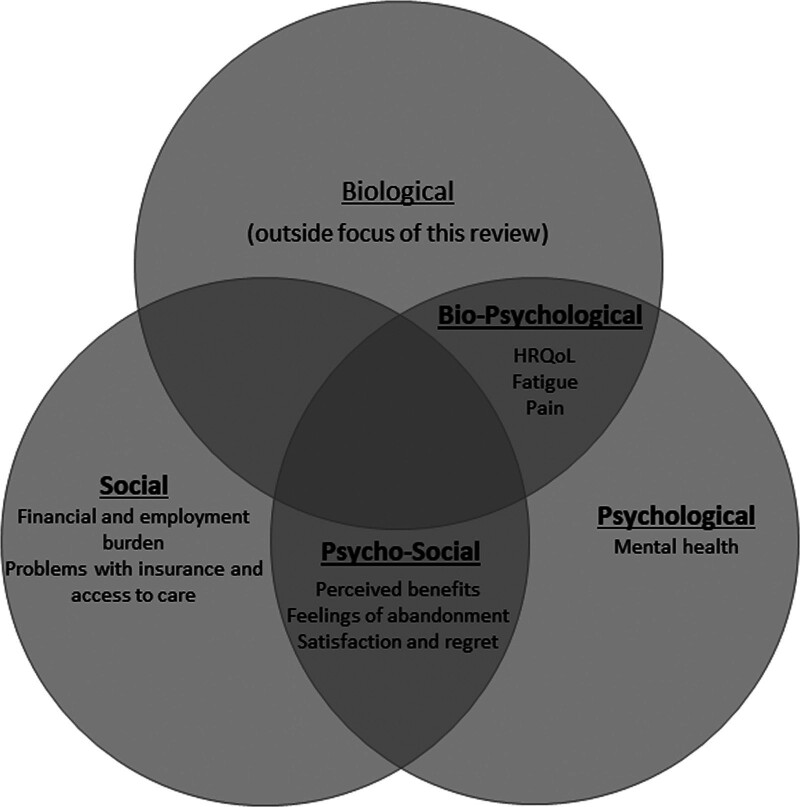
Mid- and long-term outcomes according to the biopsychosocial model.

In total, 206 articles reporting on 190 studies were included in the review. Table 1 presents aggregated data on these articles. Outcomes measured in these studies are presented according to the biopsychosocial model in Figure 1. Table 2 presents an overview of risk factors that have a significant association with mid- to long-term psychosocial outcomes in the included studies. Data extracted from each article are presented in detail in **Appendix B** (**SDC,** http://links.lww.com/TP/D77).

**TABLE 1.** Characteristics of included articles (N = 206)

Characteristic	Frequency
Country of data collection^*a*^	
USA	42
Germany	22
Netherlands	19
UK/Northern Ireland/Scotland	13
Canada	11
Turkey	9
Australia	6
Norway	6
Portugal	6
Iran	5
Sweden	4
China	4
India	3
Brazil	3
Taiwan	3
Japan	3
Switzerland	3
Poland	2
Spain	2
New Zealand	2
Korea	2
Italy	1
France	1
Nepal	1
Jordan	1
Georgia	1
Malaysia	1
Ethiopia	1
Trinidad and Tobago	1
Greece	1
Egypt	1
Article type	
Empirical study	179
Review	27
Conference report	2
Characteristics of study design	
Single center	114
Multicenter	41
Retrospective	123
Prospective	41
Characteristics of study methods	
Questionnaires	124
Interviews	31
Database analysis	9
Storytelling analysis	2
Commonly measured outcomes	
HRQoL	96
Donation experience	42
Financial/economic	32
Depression/depressive symptoms/mood	31
Anxiety	22
Pain	20
Satisfaction	17
Relationship with recipient	17
Fatigue	13
Mental health/well-being	12
Regret	10
Social support	9
Self-esteem/image	5
Coping	5
Commonly used instruments	
SF-36	77
PHQ	12
MFI-20	12
HADS	10
BDI	9
LOT	6
SWLS	6
BAI	6
SCL-90	5
SF-12	5
LDEQ	5
WHOQOL-BREF	5

a Only reported for empirical studies (excluding reviews).

BAI, Beck Anxiety Inventory; BDI, Beck Depression Inventory; HADS, Hospital Anxiety and Depression Scale; LDEQ, Living Donor Expectations Questionnaire; LOT-R, Life Orientation Test-Revised; MFI-20, Multidimensional Fatigue Inventory-20; PHQ, Patient Health Questionnaire; SCL-90, Symptom Checklist-90; SF-12, 12-Item Short Form Survey; SF-36, Short Form-36 Health Survey; SWLS, Satisfaction with life scale; WHOQOL-BREF, World Health Organization quality-of-life brief questionnaire.

**TABLE 2.** Overview of risk factors identified as significantly related to poorer psychosocial outcomes in included studies

Risk factors	Reduced HRQoL	Decreased life satisfaction	Depression/anxiety	Increased psychological symptoms/negative affect	Decreased well-being/positive affect	Poor overall donation experience/dissatisfaction/regret
Obesity/weight^10,11^	X					
Current or former smoker^12,13^	X		X			
Pain and discomfort^14–19^	X					X
Hypertension^12^			X			
Fatigue^13,20–24^	X		X			X
Longer donor recovery time^11,25–27^	X	X	X			X
Premature discharge^19^						X
Donor complications^11,26–28^	X	X		X		X
Predonation physical health problems^29^	X					
Postdonation physical health problems^20,30^						X
Poor recipient outcomes^12,19,26,28,31–40^	X		X	X	X	X
Predonation (history of) psychological symptoms^3,11,25–27,29,41,42^	X		X		X	X
Postdonation psychological symptoms^10,12,27,43^	X	X	X			X
Stress^44^				X		
Age^10,11,23,25,26,41,44,45^	X		X	X		
Sex^30,45,46^	X		X			X
Race/ethnicity^11,12^	X					X
Lack of partner/unmarried^12,44^			X	X		X
Low social support^11,44^	X				X	
No religious affiliation^44^					X	
Unemployment/earning status^44^			X		X	
Education status/level^11,12^	X		X			
Financial difficulties/burden^19,43^	X	X	X			X
Insurances difficulties^12^						X
Inadequate information^33^		X				
Decision-making effectiveness^30^						X
Moral obligation to donate^25–27^			X			
Expectation of interpersonal benefit^44^				X		
Lower appraisals of manageability^44^				X		
Avoidant coping style^44^				X		
Pessimism^33^						X
Expectations of negative health consequences after donation^44^					X	
Low positive appraisals^44^					X	
Discrepancy between expectations and actual experience^19^						X
Interference with daily lives^19^						X
Relationship to recipient^20,45,47^	X		X			

X indicates that the risk factor was investigated, and an association was found with the indicated outcome(s).

### Health-related QoL

Health-related QoL (HRQoL) is at the intersection of the biological and psychological domains as it incorporates perceived physical and mental health components. Due to the physical and psychosocial screening process, LDs generally have high HRQoL before donation.^3^ Findings on the mid-term (after 12 mo) in general suggest that physical and mental components of HRQoL have returned to baseline.^3,20,48^ Some studies report a slight decrease in perceived physical health at 12 mo postdonation,^10,49,50^ but in most cases, the level remained comparable with the general population.^10,49^ One study showed a decrease in the mental health component but no change in the physical component.^16^ In contrast, 1 study showed an improvement in mental health components at 12 mo postdonation.^51^ Findings on the long-term from numerous studies from multiple countries have shown that, on average, HRQoL is the same or better among directed donors compared with age and sex-matched individuals in the general population and with healthy (nondonor) controls.^10,11,13,14,21,22,25,28,35,36,44,45,52–75^ These findings are consistent at 10 y^76^ and 45 y postdonation,^77^ as well as for “elderly” donors older than 55/60 y.^78,79^ Similarly, reviews and meta-analyses report postdonation HRQoL to be equivalent to or higher than the general population.^2,3,47,80–87^ Some exceptions are noteworthy. Some studies report higher bodily pain compared with the general population.^82,88^ Another exception is the lower HRQoL among living unrelated compensated donors in Iran compared with the Iranian general population and nondonor nephrectomy patients.^89,90^ Experience of stressful life events and financial hardship, which motivated donation in this paid system, may help explain this difference.^91^

One factor that appears to influence HRQoL is the surgical technique. Many studies have investigated the impact of surgical techniques on HRQoL: open versus mini-incision nephrectomy,^92^ open versus laparoscopic nephrectomy,^93–99^ laparoscopic versus mini-incision donor nephrectomy,^100,101^ and hand-assisted laparoscopic nephrectomy.^102,103^ The shift from open to laparoscopic techniques has brought about improvements in HRQoL reflected in lower pain, quicker recovery, and quicker return to baseline.

Critically, however, in each LD HRQoL study, a small proportion of LDs (from 0% to 28%)—over a wide range of times postdonation—have reported a decreased HRQoL. See Table 2 for an overview of the factors that have been shown in the included studies to be significantly associated with poorer outcomes in the mid- and long-term. Reduced HRQoL is often associated with poor recipient outcomes^31,35–37^ or with donation-related complications.^104^ Standing out as important risk factors are a predonation history of psychiatric symptoms, predonation poorer physical health, a longer recovery time postdonation, greater financial burden, and medical complications for either the LD or the transplant recipient.^11,29,43^ Additional risk factors for impaired QoL, identifiable at donation, have been described. In a large Norwegian study, Mjoen et al^57^ showed that those who had doubts about donation had a lower HRQoL. Risk factors for having doubts included graft loss in the recipient, medical problems after donation, being an unrelated donor, or time since the donation of <12 y. Being poorly informed about donation may also be related to reduced satisfaction with HRQoL.^105^ After donation, life events (medical and/or psychosocial) may contribute to long-term HRQoL among LDs in the same way found among the general population.^106,107^ However, initial postdonation kidney function impairment in donors is not related to long-term reduced HRQoL.^108^

Similar to outcomes among tradition-directed donors, studies on NDDs report that most NDDs also do well in terms of HRQoL.^109–111^ However, similar to directed donors, a small percentage of NDDs have postdonation psychosocial problems.

### Fatigue and Pain

Other outcomes in the biopsychological domain are fatigue and pain. Similar to HRQoL, some studies demonstrate comparable levels of postdonation fatigue with those found in the general population.^112^ However, other studies have identified long-term fatigue^13,20–24,50^ and chronic pain^14–18,113–115^ and that these can be risk factors for reduced HRQoL.^113^ Wirken et al^20^ reported fatigue lasting 12 m postdonation; risk factors included more predonation fatigue, worse general physical functioning, and younger age. In a prospective cohort study in the United States, 12% of donors reported chronic persistent fatigue at the 24-mo follow-up.^24^ In this study, risk factors for fatigue, potentially identifiable at evaluation, were less physical activity engagement and a clinical mood disturbance. A prospective study among German donors also emphasized the relationship between predonation fatigue and stress with postdonation fatigue.^50^ There is some evidence that for a small group, fatigue may persist for up to 5 y.^56^

A systematic review suggested that parental donors experience more postoperative pain than they had expected.^116^ Pain appears to be lower among donors who underwent laparoscopic surgery compared with open surgery.^94^ In a study of 512 LDs with a mean follow-up of 6 y, Bruintjes et al^18^ reported that 5.7% of the LDs had chronic pain. Risk factors included severe early postoperative pain, previous abdominal surgery, or preexisting backache. Pain and discomfort have been shown to be related to lower donation satisfaction.^19^ Prolonged postoperative pain can be associated with delayed return to work.^117^ One study has reported that recall of postdonation pain was significantly lower among NDDs compared with directed donors and that there was a lower requirement for analgesics and lower interference with daily activities, irrespective of demographic differences.^118^

### Mental Health

In addition to studies examining the broader concept of HRQoL, which includes both physical and mental health, many studies have focused on specific aspects of the psychological domain, such as psychological symptoms, depression and anxiety, and psychological well-being. Retrospective studies generally show comparable levels of psychological complaints or psychiatric diagnoses to the general population.^119,120^ Several studies comparing trajectories of mood or psychological symptoms over time have found that fluctuations in outcomes were similar among donors and the controls.^41,121^ A study comparing LDs with individuals who had undergone nephrectomy for a benign condition found that after 5 y donors were less likely to develop depression than this matched comparator group.^42^

In general, LDs have a similar or lower rate of depression than the general population^122,123^; however, some LDs have developed depression and have related it to their donation experience.^12,25,60^ Symptoms of depression after living donation have been reported in the range of 0%–46.9%.^38,47,124,125^ Severe depression has been reported in the range of 4%–22%.^69,126,127^ Lentine et al^32^ reported a depression diagnosis among 4.2% at 1 y and 11.5% at 5 y. Symptoms of anxiety after donation have been reported in the range of 0%–67%.^38,47,126,127^ Severe anxiety has been reported among 0.6%–9.2% of donors.^21,69,124,128^ One study of Chinese donors reported significantly higher postdonation anxiety than the average level in the general population.^70^ It is important to differentiate between studies that report a clinical diagnosis of depression/anxiety compared with those reporting symptoms based on screening instruments. Among the latter, the level of false positives is likely to be high, given the nature of screening instruments. Estimates also vary according to setting, instruments used, and time since donation.

Few studies have investigated personality traits or the psychological profile of living kidney donors. One German study demonstrated higher than average agreeableness in donors,^112^ which is characterized by being good-natured, cooperative, and prosocial. Agreeableness was also significantly correlated with lower anxiety, depression, and fatigue. An assessment of early maladaptive schemas among kidney and liver donors found that elevated “pessimism,” “social-isolation,” and “emotional deprivation” schemas were associated with higher levels of depression postdonation. The authors highlighted the potential importance of social support in the prevention of depressive symptoms among donors.^129^

Several demographic and psychosocial factors, identified before and after donation, significantly impact psychological symptoms (see Table 2). Risk factors for anxiety and depression included lack of college education, not being married or living with a partner, being a smoker, and having hypertension.^12,38,44^ ^38^ Lentine et al^32^ studied 4650 LDs and found an increased risk of depression in nonspousal, biologically unrelated LDs associated with their recipient’s death or graft loss; however, the same association was not seen in biologically related LDs. Parent donors have been shown to report more symptoms of depression and anxiety than sibling donors.^47^ Some of the first studies in this area identified the link between an unsuccessful outcome for the recipient and greater psychological distress in the donor.^39,40^ Further cross-sectional and retrospective studies have also demonstrated a relationship between poor recipient outcomes and elevated donor emotional response, depression, and anxiety.^31,130,131^ In a prospective study in the Netherlands, Timmerman et al^28^ studied LDs predonation and then at 3 and 12 mo postdonation. An increase in LD psychological symptoms was associated with both LD complications and recipient rehospitalizations. Recipient rehospitalizations were also associated with decreased LD well-being, although this relationship became weaker over time. Furthermore, psychological factors, such as coping style and appraisals of manageability, were associated with mental health at all time points.^44^ A systematic review confirmed that predonation mental health is strongly linked to postdonation mental health.^38^ Timmerman et al^28^ recommended that centers review the LD profile before donation and based on the number and magnitude of the identified risk factors, determine which LDs might need more postdonation support.

It is noticeable that many studies were conducted in Western countries; however, there is an emerging literature on mid- to long-term psychological outcomes after directed donation in developing countries (see Table 1). One such study in Ethiopia showed that 30% of donors scored in the lowest quartile for psychological well-being.^132^

Studies among NDDs that have investigated psychological symptoms after donation have found similar results to studies among directed donors. For example, in a series of studies in the Netherlands among NDDs, there was an overall increase in postdonation psychological symptoms, but the mean score remained within the average range of the general population.^133,134^ Moreover, in a cross-sectional analysis among this group psychological well-being was higher than in the general population.^135^ Maple et al^109^ used a series of validated questionnaires in comparing 110 NDDs with 80 directed donors and found no difference between groups in current depression, anxiety, stress, self-esteem, or well-being. Wadstrom et al^136^ studied 24 NDDs in Sweden and found that of these 24 NDDs, 46% reported increased self-esteem after donation, and 33% reported increased happiness after donation. Jacobs et al^137^ surveyed 77 NDDs and reported that only a few felt that the donation experience had been very or extremely stressful. The 2 major stressors were physical consequences and fear of the recipient rejecting the transplant. The majority of respondents felt there had been either improvement or no change in their life since donation; the majority felt an increased appreciation of life and increased self-worth. A small percentage said their perceived physical health or mental health was a little worse. Given that the questionnaire was administered many years postdonation, these responses may have been unrelated to donation, but due to other life-related events (eg, aging). One study on Israeli NDDs found higher death anxiety but lower psychological distress compared with the control population.^138^ With the rise in the use of social media to find a LD, coercion has been a topic of study; however, no difference was found in a study comparing traditional NDDs and community-solicited NDDs.^111^ More research is needed on these emerging subgroups of NDDs.^139^

Qualitative studies among NDDs have identified major themes associated with psychosocial outcomes after donation.^140-142^ The support that NDDs receive from their social networks appears to influence the experience of donation. Many face a lack of emotional support.^143^ Some report interpersonal stress when their partner or other family members, such as adult children, do not support the donation.^140^ This sometimes leads to “uneasy negotiations with others”^142^ and requires persistence, patience, and determination.^140,141^ During this period, most donors felt like their lives were on hold while waiting for test results and the final approval.^140,142^ Not dissimilar to directed donors, when actually admitted for nephrectomy NDDs report the “paradox of being an unobvious patient,” a healthy individual undergoing an operation and needing care but feeling uncomfortable about this.^142^ In some cases, this led to needs not being met and dissatisfaction (see below). With regard to anonymity, the large majority supported and were satisfied with this approach to nondirected donation, although there is an appreciation of anonymous correspondence and feedback on the recipient outcomes.^140,141^

### Benefits of Living Donation

At the intersection of psychological and social outcomes are the benefits of living donation, feelings of abandonment and unmet needs, and satisfaction and regret. Most studies after living donation have focused on risk factors for poor outcome; however, many authors also discuss the benefits of living donation.^33,34,144-155^ Many LDs feel good about donation and have significantly improved self-esteem.^102,116,136,156^ Rodrigue et al^34^ found that among LDs, there were differing postdonation trajectories leading to interpersonal benefit, and separately, intrapersonal benefit (personal or spiritual growth). Those whose recipients were doing well benefited the most. Those having exceedingly high expectations about recipient outcomes were at risk for less benefit. The authors recommended identifying LD candidates with unreasonable expectations of recipient benefit and addressing them before donation. Another study comparing posttraumatic growth among with donors to healthy controls, cancer survivors, and traffic accident survivors found a higher positive change in relationships and self-perception among donors but a relatively lower change in life philosophy, which is in line with the idea that donation fits donors’ life view or principles.^157^ Sex differences in benefits have received little attention and findings are mixed. One study found that more female donors reported donations having a positive influence on their lives than male donors,^46^ whereas another found no sex differences.^158^

Findings may depend on the methodology used, for example, most studies demonstrating benefits are retrospective. Prospective studies fail to find statistically significant increases in positive (generic) outcomes.^48,121^ Positive themes have emerged repeatedly from qualitative studies: a general sense of satisfaction and empowerment through donation has been repeatedly reported.^140,141,159^ In some cases, this was described as benefits gained, such as a healthier lifestyle and the knowledge that they had contributed to society resulting in fulfillment and peace.^141^ Others report this in terms of self-actualization and meaning-making.^142^ There is evidence for stability in body image and body esteem over time among donors.^160^

Benefits experienced may also depend on the relationship with the recipient. Many studies report either no change or more intense emotional bonds with the recipient^63^ or within the family.^167^ Studies among parental donors showed that the donor felt the relationship with the child had improved after the operation.^116,168^ LDs who were in an interdependent relationship with their recipient (defined as sharing a household with the recipient, or having significant caregiving activities for the recipient) reported that LD benefits were related to health and well-being (eg, reduced stress), time and finances (recipient’s return to work, ability to travel), and interpersonal relationships.^169^ Two studies have investigated marital satisfaction among spousal versus nonspousal donors with contradicting findings.^170,171^ It is possible that spouses who are content in their marriage are more likely to donate and concurrently that having a chronically ill partner negatively influences marital satisfaction. With regard to the impact of direct versus indirect donation, a comparative study highlighted no significant differences between “chain” and traditional donors on benefits, such as interpersonal benefit and personal growth.^172^

Generic measures may not be sensitive of relevant enough to capture donation-related benefits and qualitative studies may be better placed to capture the meaning of donation. Moreover, qualitative studies among donors demonstrate more nuances in the findings regarding the impact on QoL and often portray the simultaneous combination of positive and negative aspects.^159,173,174^ One qualitative study reported tensions under the surface within the family.^175^ Of course, donation does not offer protection against the disintegration of a relationship and marital conflicts have also been reported postoperatively.^176^ Indeed, a synthesis of the meaning of living donation highlighted themes indicating benefits (such as growth and pride) alongside themes highlighting donor struggles (such as loneliness and abandonment).^177^ In a comprehensive review, Tong et al^147^ reviewed qualitative studies of experiences after donation and reported on both positive and negative experiences. The authors concluded, “kidney donation has a profound and multifaceted impact on the lives of donors.”^147^ These findings have been replicated with both positive and negative themes being reported in qualitative studies. Examples of themes implying benefits include donation as empowering for health (eg, feeling vulnerable after donation, adopting healthier lifestyles), overall happiness, and relief after surgery.^145,178^ Seeing the recipient’s improvement in health and justifying the experience were also commonly reported themes.^22,178^ Others recommend seeking information to promote a comprehensive understanding of the process alongside building a community for support.^179^

### Donation Satisfaction and Regret

Overall, LDs have self-reported a low rate of regret about donation, both among directed donors^12,20,26,30,46,48,60,70,110,117,127,140^ and NDDs.^133,136,137,140^ In a long-term follow-up of Swedish donors, <1% reported regret^180^ although in long-term follow-up study of German donors, 24% had some form of regret.^112^ Of the 190 participants in a UK study, 3.7% of NDDs versus 7.5% of directed donors reported regretting their decision to donate.^109^ Similarly, results from the WHOLE-donor study reported 2.1% regret after donation among >800 donors^12^ with no significant difference between directed and NDD groups. Among compensated Iranian donors regret has also been found to be low.^185^ Risk factors for regret include being African American, being more likely to have trouble obtaining or changing life insurance, and being less likely to be married or living with a partner^12^ (see Table 2). Qualitative findings from various countries support the lack of regret reported among LDs.^140,141,143,178^

Closely linked to (lack of) regret is satisfaction with the donation. Overall, the level of satisfaction is reported to be high.^19,33,94,133^ Some describe it as being a “worthwhile experience”^191^ or a good overall experience.^192,193^ Menjivar et al^19^ studied satisfaction with donation and conceptualized satisfaction as a multifaceted construct, rather than being addressed in a single area. Overall, LDs had a high level of satisfaction, and any dissatisfaction was related to discrepancies between expectations and actual experiences, interference of donation on daily activities, and pain and discomfort. The authors found that those with lower satisfaction were more likely to report that hospital discharge was premature, had economic losses, or felt their recipient had worse outcomes. Similarly, Kobayashi et al,^33^ using a Japanese version of a satisfaction scale that encompassed the entire donation experience, reported lower satisfaction was related to a perception of receiving inadequate information, donor pessimism, and recipient outcome (higher recipient creatinine levels). Conversely, adequate information provision and proactive professional support has been reported among donors who viewed donation positively.^194^ Poorer recipient outcomes have been repeatedly related to lower satisfaction.^65,117,188^ There is a plethora of evidence that almost all donors would make the same decision again if they could, which underscores their decisional satisfaction^46,105,114,195,196^ and would recommend donation to others.^66,67,70,125,197^

Similarly, a recent review of qualitative research among NDDs also highlights the perceived benefits of empowerment and satisfaction.^143^ In this review, a sense of connectedness was also reported as a benefit to family members, their social network, and the community. For NDDs, this connection is deepened when they receive some kind of anonymous response from the recipient,^141,142^ whereas a lack of (anonymous) response appears to be linked to greater dissatisfaction.^133,140,142,143^

### Feelings of Abandonment and Unmet Needs

Despite the return to predonation levels of HRQoL and lack of regret, some living kidney donors report some gaps in the care and unmet needs. In an interview study conducted by Manera et al^145^ in Canada and Australia, one of the 4 major themes identified was “neglect and inattention” (eg, hospital abandonment, lack of concern with prolonged convalescence).

Studying Jordanian donors, Al Breizat^173^ also reported similar feelings of being forgotten after donation. Among NDDs in the Netherlands, “Ongoing negative emotions” appear to be related to unmet needs, dissatisfaction about the process, and reimbursement challenges.^159^ A synthesis of 40 qualitative studies also supported the idea that donors “desire attention” in the form of recognition for their donation act and that sometimes attention directed toward the recipient can be resented.^163^ Manera et al^145^ recommended ongoing access to healthcare, psychosocial support, and education.

In the overall context of hospitalized surgical patients, LDs are well; and when admitted to a floor with other surgical patients, it may be the case that LDs need the least attention. However, LDs require the same treatment as all other postoperative patients including the necessary attention and pain management. Similarly, some LDs have ongoing problems for longer than the standard expected 6-wk recovery time. Not all transplant programs may be prepared to deal with these problems, leaving the LDs feeling abandoned. One study highlighted the unmet need for greater psychosocial follow-up by the healthcare team.^198^ These issues were emphasized at a consensus conference on LD follow-up, at which inadequate care of LDs with long-term medical or psychosocial consequences of donation was discussed in detail.^146^ Key recommendations included developing long-term follow-up studies to identify the types and frequency of problems and addressing care of the LDs with these long-term consequences.

### Financial and Employment Burden

In the social domain, the donor-specific outcomes of financial and employment burden and problems with insurance and access to care were reported on in the included studies. In many countries, the cost of the LD evaluation and the surgery is paid by a universal healthcare system or by the recipient’s healthcare insurance. However, some LDs incur direct costs (ie, travel, hotel, meals) for both the evaluation and then again for the surgery.^63,165,199^ In the United States, where there is no universal healthcare system, some LDs also have medical expenses related to the donation itself.^200^ Direct costs can amount to a considerable sum, which takes its toll on both well-being^38,201^ and finances.^191,199^ In addition, LDs have indirect costs (ie, lost wages while having daily/monthly expenses [eg, mortgage, rent, groceries]).^63,165,181,199,202^ Indirect costs are rarely covered by the employer or reimbursement policies.^203^

Estimates of costs and financial burden vary. Prospective studies from Canada and the United States found that one-third of LDs have significant expenses that often exceed the cap put on reimbursement policies.^204,205^ Clarke et al^206^ reviewed 35 retrospective studies from 12 countries; each study revealed financial expenses to the LD associated with the process of donation. Another review found that 80% of donors experienced financial loss.^207^ Costs can be a considerable burden for many LDs and deter patients from discussing living donation and eligible candidates from actually donating.^208^ In addition, in countries without universal healthcare, many LD candidates have been uninsured and are worried about postdonation complications and their costs.

It has been legal in most countries for recipients to help pay LD costs. However, end-stage kidney disease disproportionally affects the poor; and LDs and their recipients are commonly in the same financial stratum, resulting in recipients not having the ability to reimburse LDs.

Although expenses have been described, there has been little description of the burden felt by the LDs. Direct expenses or lost income may or may not be a burden depending on numerous personal factors, including income, savings, debts, benefits, and other resources. Economic burden has been reported from countries with and without universal healthcare.^57,200,205^ In an Indian study, 87% of donors reported receiving financial support from the recipient or family members, which may be indicative of financial barriers to donation in this setting.^192^ In a multicenter Korean study, there was evidence that in the 2 y postdonation, donors have increased chances of being unemployed or losing their job, as well as higher odds of deterioration in financial situation.^209^ A multicenter study of LDs surveying a mean 17 y postdonation (range, 5–48 y) reported that 20% of LDs felt that donation had been associated with a financial burden.^26^ Among donors claiming reimbursement through the Donor Shield program, 81% reported that a lack of reimbursement would have led to financial hardship.^210^ Rodrigue et al^205^ created an empirically calculated, externally determined, description of the burden by calculating net financial loss (ie, total costs of LDs and caregivers minus net financial assistance) divided by monthly household income. Using this definition, they determined that “burden” was highest in those with greater travel distance, lower household income, and more unpaid work hours missed. This calculation provides a reproducible definition of financial impact but does not account for the subjective experience of financial burden—an experience that might be affected by the degree to which one lives from paycheck to paycheck, has financial dependents, has other family medical expenses or debt, or is in seasonal work.

Financial burden may also influence return to work; factors influencing return to work have been found to vary among men and women.^211^

Many countries (eg, Canada, Australia, Singapore, Israel, the Netherlands) have developed government-sponsored programs to reimburse donor costs and to provide some support for lost wages. In the United States, transplant programs and patient advocacy groups have lobbied Congress for financial support to provide financial neutrality for donation, and recently there has been progress in moving forward to achieve this.^212-214^ Whereas many elements of financial neutrality are easy to define, others are more difficult. In countries with universal healthcare, long-term postdonation expenses are less of an issue. However, in countries without universal healthcare, what should be included in long-term reimbursement is more challenging to define, as directly correlating a medical expense to a distant past kidney donation may be less clear. Guidelines have been proposed regarding what should and should not be covered in a program designed for true financial neutrality.^214,215^

### Problems With Insurance and Access to Care

In countries with universal healthcare, the costs of long-term donor care are generally less of a problem. In contrast, in countries such as the United States that do not have universal healthcare, the provision of long-term care may be an issue for some donors. Historically, in the United States, 12%–16% of LDs were uninsured at the time of donation.^216,217^ In 2015, after the enactment of the Affordable Care Act, about 9% were uninsured.^218^ LDs of color and LDs with additional health risk factors at the time of evaluation were more likely to lack access to routine healthcare or financial resources needed for donation.

In addition, in countries with and without universal healthcare, LDs have experienced problems with insurability (for both health and life insurance) or have incurred higher insurance premiums postdonation.^26,57,219,220^ A systematic review on this issue found that although health insurance companies generally reported no barriers or increased fees for donors’ health, disability, or life insurance, donors themselves did encounter insurance issues.^221^ Boyarsky et al^219^ found that of those who changed or initiated life insurance postdonation, 25% experienced difficulty.^219^ Of the 2455 LDs in the RELIVE study, 2.1% were denied life insurance after donation.^26^

## LIMITATIONS TO THE CURRENT EVIDENCE

There are several limitations to studies on mid- to long-term LKD psychosocial outcomes. First, similar to the studies on long-term medical outcomes, the studies on psychosocial outcomes typically have not compared donors with well-matched control groups (those meeting donation criteria but not donating). Matched comparator groups are required to allow meaningful interpretation of outcomes among donors. Second, most of the long-term data are retrospective and have been provided by a small number of groups in mostly single-center studies. Third, there is a lack of standardization in outcome measures, in particular, donation-specific outcomes are largely measured using self-developed nonvalidated measures. Development and validation of donor-specific measures would help facilitate the comparison of these outcomes. Fourth, regarding reporting standards, we noted that 14% (30/206) of articles failed to report the length of follow-up since donation. Fifth, the vast majority of LDs in these studies have been conducted in Western countries. There is increasing attention to the disparity in access to living donation^222^ and greater knowledge on psychosocial outcomes among donors in diverse populations and settings, which can help contribute to optimizing education on living donation. Sixth, LD acceptance criteria have become less restrictive over time. Compared with 5 decades ago, LDs are older, are more likely to have obesity or have hypertension at the time of donation, and have a greater comorbidity burden.^223-225^ Despite this expansion of criteria to date, there does not appear to have been a change in impact on psychosocial outcomes; however, this requires monitoring over time. Seventh, despite identifying a subgroup of donors with suboptimal psychosocial outcomes, there is a noticeable lack of interventions targeted at improving these outcomes. A limited number of studies examine the short-term impact of behavioral interventions^226,227^; however, none were found with a follow-up of ≥12 mo. Finally, to date, there are a limited number of studies on the postdonation outcomes for LDs participating in more recent approaches designed to increase the number of recipients who can receive an LD kidney, including donation through biological barriers (ABOi and HLAi) and advanced donation. There may be different risks, particularly psychosocial risks, for each of these groups and each needs to be studied. Without identifying and studying subgroups that are potentially at increased risk (versus controls), we will not understand the true risk for LDs at a more individualized level. Moreover, the justification of some of these transplantations, in particular HLA-incompatible transplantation, is a presumed increase in QoL; however, this has yet to be demonstrated.^228^

## CONCLUSIONS

Overall, psychosocial outcomes are positive for the majority of donors. This research area has developed considerably since the 1990s when the focus was largely on HRQoL to compare surgical procedures. In recent years, there has been increasing interest in understanding the factors contributing to differences in mid- and long-term psychosocial outcomes. Quantitative studies have been enriched by qualitative studies, which have added a greater understanding of the lived experience. These studies highlight that living donation is complex and multifaceted and that the experience almost always characterized by both positives and negatives. Almost all studies also identify a small group of donors who fare less well with regard to mid- and long-term psychosocial outcomes. Risk factors for reduced HRQoL identifiable at evaluation include preexisting psychological problems (low psychological function or mood disorders), ambivalence toward donation, lack of support, younger age, unrealistic expectations, reduced physical functioning/vitality, and inadequate information. These factors need to be considered at the time of donor evaluation, counseling, and acceptance. Moreover, if an increased-risk candidate is accepted for donation, there should be a plan for increased pre- and postdonation support. The fact that “inadequate information” is a risk factor should be a stimulus for all transplant programs to review their donor education processes.

Postdonation risk factors for reduced HRQoL include donor complications/medical problems, longer recovery, recipient complications/graft loss, greater financial burden, persistent fatigue, and chronic pain. There needs to be recognition that if these problems develop, the donor needs additional support. As noted above, some donors do well and feel that recurring follow-up is unnecessary and intrusive. In contrast, those who need support and do not get it feel abandoned. Importantly, this review highlights that ongoing care should extend beyond the first year. The finding that donation-related events can affect mental and perceived physical health should prompt centers to identify and develop a care plan for those at risk, to consider access to care and methods of providing it, and to align LDs’ expectations with likely outcomes for both themselves and their recipient.

The findings highlighted methodological improvements needed in the area of LD research. Specifically, there is a need for matched comparator groups and prospective long-term follow-up beyond 12 mo. Agreeing standardized donation-specific outcomes and outcome measures would also benefit the comparison of data over time and across settings. Finally, this review contributes to the literature by identifying 3 areas requiring immediate attention from the transplant community in both research and clinical care: (1) recognizing and providing care for the minority of LDs who have long-term physical and psychosocial issues relating to donation, (2) minimizing donation-related financial burden, and (3) studying interventions to minimize long-term problems.

## ACKNOWLEDGMENTS

The authors thank the following people for their contribution: Stephanie Taylor for administrative assistance in preparation of the article; Crista Niehot from the Erasmus MC Medical Library for developing and updating the search strategies; Jaden Blazier for assistance with data extraction; and Liset Pengel for methodological pointers.

## Supplementary Material



## References

[1] Hariharan S Israni AK Danovitch G. Long-term survival after kidney transplantation. N Engl J Med. 2021;385:729–743. 34407344 10.1056/NEJMra2014530

[2] Clemens KK Thiessen-Philbrook H Parikh CR; Donor Nephrectomy Outcomes Research (DONOR) Network. Psychosocial health of living kidney donors: a systematic review. Am J Transplant. 2006;6:2965–2977. 17294524 10.1111/j.1600-6143.2006.01567.x

[3] Wirken L van Middendorp H Hooghof CW. The course and predictors of health-related quality of life in living kidney donors: a systematic review and meta-analysis. Am J Transplant. 2015;15:3041–3054. 26414703 10.1111/ajt.13453

[4] Engel GL. The need for a new medical model: a challenge for biomedicine. Science. 1977;196:129–136. 847460 10.1126/science.847460

[5] Wade DT Halligan PW. The biopsychosocial model of illness: a model whose time has come. Clin Rehabil. 2017;31:995–1004. 28730890 10.1177/0269215517709890

[6] Maggiore U Budde K Heemann U; ERA-EDTA DESCARTES Working Group. Long-term risks of kidney living donation: review and position paper by the ERA-EDTA DESCARTES working group. Nephrol Dial Transplant. 2017;32:216–223. 28186535 10.1093/ndt/gfw429

[7] Matas AJ Rule AD. Long-term medical outcomes of living kidney donors. Mayo Clin Proc. 2022;97:2107–2122. 36216599 10.1016/j.mayocp.2022.06.013 PMC9747133

[8] Matas AJ Garvey CA Jacobs CL. Nondirected donation of kidneys from living donors. N Engl J Med. 2000;343:433–436. 10933745 10.1056/NEJM200008103430611

[9] Dor FJMF Massey EK Frunza M. New classification of ELPAT for living organ donation. Transplantation. 2011;91:935–938. 21423070 10.1097/TP.0b013e3182129236

[10] Klop KWJ Timman R Busschbach JJ. Multivariate analysis of health-related quality of life in donors after live kidney donation. Transplant Proc. 2018;50:42–47. 29407329 10.1016/j.transproceed.2017.10.019

[11] Gross CR Messersmith EE Hong BA; RELIVE Study Group. Health-related quality of life in kidney donors from the last five decades: results from the RELIVE study. Am J Transplant. 2013;13:2924–2934. 24011252 10.1111/ajt.12434 PMC4091665

[12] Holscher CM Leanza J Thomas AG. Anxiety, depression, and regret of donation in living kidney donors. BMC Nephrol. 2018;19:218. 30180815 10.1186/s12882-018-1024-0 PMC6122576

[13] de Groot IB Stiggelbout AM van der Boog PJM; PARTNER-Study Group. Reduced quality of life in living kidney donors: association with fatigue, societal participation and pre-donation variables. Transpl Int. 2012;25:967–975. 22780196 10.1111/j.1432-2277.2012.01524.x

[14] Rodrigue J Vishnevsky T Fleishman A. Patient-reported outcomes following living kidney donation: a single center experience. J Clin Psychol Med Settings. 2015;22:160-168. 26123551 10.1007/s10880-015-9424-9 PMC4575847

[15] Fleishman A Khwaja K Schold JD; KDOC Study Group. Pain expectancy, prevalence, severity, and patterns following donor nephrectomy: findings from the KDOC Study. Am J Transplant. 2020;20:2522–2529. 32185880 10.1111/ajt.15861 PMC7483675

[16] Smith GC Trauer T Kerr PG. Prospective psychosocial monitoring of living kidney donors using the Short Form-36 health survey: results at 12 months. Transplantation. 2004;78:1384–1389. 15548979 10.1097/01.tp.0000140967.34029.f1

[17] Zorgdrager M van Londen M Westenberg LB. Chronic pain after hand-assisted laparoscopic donor nephrectomy. Br J Surg. 2019;106:711–719. 30919435 10.1002/bjs.11127 PMC6593841

[18] Bruintjes MHD van Helden EV de Vries M. Chronic pain following laparoscopic living-donor nephrectomy: prevalence and impact on quality of life. Am J Transplant. 2019;19:2825–2832. 30868731 10.1111/ajt.15350 PMC6790588

[19] Menjivar A Torres X Paredes D. Assessment of donor satisfaction as an essential part of living donor kidney transplantation: an eleven-year retrospective study. Transplant Inter. 2018;31:1332–1344. 10.1111/tri.13334 30144365

[20] Wirken L van Middendorp H Hooghof CW. Psychosocial consequences of living kidney donation: a prospective multicentre study on health-related quality of life, donor-recipient relationships and regret. Nephrol Dial Transplant. 2019;34:1045–1055. 30544241 10.1093/ndt/gfy307

[21] Sommerer C Feuerstein D Dikow R. Psychosocial and physical outcome following kidney donation-a retrospective analysis. Transpl Int. 2015;28:416–428. 25557158 10.1111/tri.12509

[22] Meyer K Wahl AK Bjørk IT. Long-term, self-reported health outcomes in kidney donors. BMC Nephrol. 2016;17:8. 26754798 10.1186/s12882-016-0221-y PMC4709885

[23] Sommerer C Estelmann S Metzendorf NG. Gender disparity in health-related quality of life and fatigue after living renal donation. BMC Nephrol. 2018;19:377. 30587146 10.1186/s12882-018-1187-8 PMC6307222

[24] Rodrigue JR Fleishman A Schold JD; KDOC Study Group. Patterns and predictors of fatigue following living donor nephrectomy: findings from the KDOC Study. Am J Transplant. 2020;20:181–189. 31265199 10.1111/ajt.15519

[25] Jowsey SG Jacobs C Gross CR; RELIVE Study Group. Emotional well-being of living kidney donors: findings from the RELIVE Study. Am J Transplant. 2014;14:2535–2544. 25293374 10.1111/ajt.12906 PMC4205186

[26] Jacobs CL Gross CR Messersmith EE; RELIVE Study Group. Emotional and financial experiences of kidney donors over the past 50 years: the RELIVE Study. Clin J Am Soc Nephrol. 2015;10:2221–2231. 26463883 10.2215/CJN.07120714 PMC4670771

[27] Messersmith EE Gross CR Beil CA; RELIVE Study Group. Satisfaction with life among living kidney donors: a RELIVE Study of long-term donor outcomes. Transplantation. 2014;98:1294–1300. 25136843 10.1097/TP.0000000000000360 PMC4333130

[28] Timmerman L Laging M Timman R. The impact of the donors’ and recipients’ medical complications on living kidney donors’ mental health. Transplant Inter. 2016;29:589–602. 10.1111/tri.12760 26895841

[29] Wirken L van Middendorp H Hooghof CW. Combining transplant professional’s psychosocial donor evaluation and donor self-report measures to optimise the prediction of HRQoL after kidney donation: an observational prospective multicentre study. BMJ Open. 2022;12:e045249. 10.1136/bmjopen-2020-045249 PMC8895930 35236728

[30] Liu KL Chien CH Hsieh CY. Effective decision-making and decisional regret in living kidney donors of Taiwan. Transplant Proc. 2018;50:3059–3064. 30577166 10.1016/j.transproceed.2018.08.053

[31] Shi Y Zhang H Nie Z. Quality of life, anxiety and depression symptoms in living related kidney donors: a cross-sectional study. Int Urol Nephrol. 2023;55:2335–2343. 36881268 10.1007/s11255-023-03542-z

[32] Lentine KL Schnitzler MA Xiao H. Depression diagnoses after living kidney donation: linking U.S. Registry data and administrative claims. Transplantation. 2012;94:77–83. 22691958 10.1097/TP.0b013e318253f1bc PMC4447542

[33] Kobayashi S Akaho R Omoto K. Post-donation satisfaction in kidney transplantation: a survey of living donors in Japan. BMC Health Serv Res. 2019;19:755. 31655578 10.1186/s12913-019-4556-5 PMC6815382

[34] Rodrigue JR Paek M Whiting J. Trajectories of perceived benefits in living kidney donors: association with donor characteristics and recipient outcomes. Transplantation. 2014;97:762–768. 24342981 10.1097/01.TP.0000437560.23588.ef

[35] Westlie L Fauchald P Talseth T. Quality of life in Norwegian kidney donors. Nephrol Dial Transplant. 1993;8:1146–1150. 7818616

[36] Johnson EM Najarian JS Matas AJ. Living kidney donation: donor risks and quality of life. Clin Transpl. 1997;231:240. 9919408

[37] Watson JM Behnke MK Fabrizio MD. Recipient graft failure or death impact on living kidney donor quality of life based on the living organ donor network database. J Endourol. 2013;27:1525–1529. 24134317 10.1089/end.2013.0189

[38] Ong JQL Lim LJH Ho RCM. Depression, anxiety, and associated psychological outcomes in living organ transplant donors: a systematic review. Gen Hosp Psychiatry. 2021;70:51–75. 33721612 10.1016/j.genhosppsych.2021.03.002

[39] Hirvas J Enckell M Kuhlback B. Psychological and social problems encountered in active treatment of chronic uraemia. II.The living donor. Acta Med Scand. 1976;200:17–20. 785956 10.1111/j.0954-6820.1976.tb08189.x

[40] Hirvas J Enckell M Kuhlback B. Psychological and social problems encountered in active treatment of chronic uraemia. III. Prediction of the living donor’s psychological reaction. Acta Med Scand. 1980;208:285–287. 7004093 10.1111/j.0954-6820.1980.tb01195.x

[41] Rodrigue JR Schold JD Morrissey P; KDOC Study Group. Mood, body image, fear of kidney failure, life satisfaction, and decisional stability following living kidney donation: findings from the KDOC study. Am J Transplant. 2018;18:1397–1407. 29206349 10.1111/ajt.14618 PMC5988866

[42] Tomer N Small A Mirza O. Evidence of resilience in kidney donors: a New York statewide cohort analysis. Transplant Proc. 2021;53:803–807. 33551185 10.1016/j.transproceed.2021.01.021

[43] Chen KH Yeh LC Huang HL. Factors determining physical and mental quality of life of living kidney donors in Taiwan. Transplant Proc. 2016;48:745–748. 27234727 10.1016/j.transproceed.2015.12.060

[44] Timmerman L Timman R Laging M. Predicting mental health after living kidney donation: the importance of psychological factors. Br J Health Psychol. 2016;21:533–554. 26791347 10.1111/bjhp.12184

[45] de Graaf Olson W Bogetti-Dumlao A. Living donors’ perception of their quality of health after donation. Prog Transplant. 2001;11:108–115. 11871045 10.1177/152692480101100206

[46] von Zur-Muhlen B Yamamoto S Wadstrom J. Few gender differences in attitudes and experiences after live kidney donation, with minor changes over time. Ann Transplant. 2017;22:773–779. 29284769 10.12659/AOT.906129 PMC6248298

[47] Pawłowski M Fila-Witecka K Rymaszewska JE. Quality of life, depression and anxiety in living donor kidney transplantation. Transplant Rev. 2020;34:100572. 10.1016/j.trre.2020.100572 33038784

[48] Maple H Chilcot J Weinman J. Psychosocial wellbeing after living kidney donation—a longitudinal, prospective study. Transpl Int. 2017;30:987–1001. 28445627 10.1111/tri.12974

[49] Lumsdaine JA Wray A Power MJ. Higher quality of life in living donor kidney transplantation: prospective cohort study. Transpl Int. 2005;18:975–980. 16008749 10.1111/j.1432-2277.2005.00175.x

[50] Suwelack B Berger K Wolters H; SoLKiD Study Group. Results of the prospective multicenter SoLKiD cohort study indicate bio-psycho-social outcome risks to kidney donors 12 months after donation. Kidney Int. 2022;101:597–606. 34953772 10.1016/j.kint.2021.12.007

[51] Fathallah MG Fadel FI Saadi GE. Renal outcome and health related quality of life of living related donors in pediatric kidney transplantation. Biomed Pharmacol J. 2021;14:1397–1403.

[52] Menjivar A Torres X Manyalich M. Psychosocial risk factors for impaired health-related quality of life in living kidney donors: results from the ELIPSY prospective study. Sci Rep. 2020;10:21343. 33288792 10.1038/s41598-020-78032-8 PMC7721886

[53] Schover LR Streem SB Boparai N. The psychosocial impact of donating a kidney: long-term followup from a urology based center. J Urol. 1997;157:1596–1601. 9112484 10.1016/s0022-5347(01)64803-1

[54] Johnson EM Anderson JK Jacobs C. Long-term follow-up of living kidney donors: quality of life after donation. Transplantation. 1999;67:717–721. 10096528 10.1097/00007890-199903150-00013

[55] Isotani S Fujisawa M Ichikawa Y. Quality of life of living kidney donors: the short-form 36-item health questionnaire survey. Urology. 2002;60:588–592. 12385912 10.1016/s0090-4295(02)01865-4

[56] Dols LF Ijzermans JN Wentink N. Long-term follow-up of a randomized trial comparing laparoscopic and mini-incision open live donor nephrectomy. Am J Transplant. 2010;10:2481–2487. 20977639 10.1111/j.1600-6143.2010.03281.x

[57] Mjøen G Stavem K Westlie L. Quality of life in kidney donors. Am J Transplant. 2011;11:1315–1319. 21486387 10.1111/j.1600-6143.2011.03517.x

[58] Dew MA Jacobs CL. Psychosocial and socioeconomic issues facing the living kidney donor. Adv Chronic Kidney Dis. 2012;19:237–243. 22732043 10.1053/j.ackd.2012.04.006 PMC3384485

[59] Janki S Klop KW Dooper IM. More than a decade after live donor nephrectomy: a prospective cohort study. Transpl Int. 2015;28:1268–1275. 25865340 10.1111/tri.12589

[60] Krishnan N Mumford L Lipkin G. Comparison of medium-term outcomes of living kidney donors with longitudinal healthy control in the United Kingdom. Transplantation. 2020;104:e65–e74. 31895342 10.1097/TP.0000000000003082

[61] Lima DX Petroianu A Hauter HL. Quality of life and surgical complications of kidney donors in the late post-operative period in Brazil. Nephrol Dial Transplant. 2006;21:3238–3242. 16921185 10.1093/ndt/gfl433

[62] Maglakelidze N Pantsulaia T Managadze L. Assessment of health-related quality of life in living kidney donors. Transplant Proc. 2011;43:373–375. 21335225 10.1016/j.transproceed.2010.12.016

[63] Giessing M Reuter S Schonberger B. Quality of life of living kidney donors in Germany: a survey with the validated Short Form-36 and Giessen Subjective Complaints List-24 questionnaires. Transplantation. 2004;78:864–872. 15385806 10.1097/01.tp.0000133307.00604.86

[64] Padrao MB Sens YA. Quality of life of living kidney donors in Brazil: an evaluation by the short form-36 and the WHOQOL-bref questionnaires. Clin Transplant. 2009;23:621–627. 19664019 10.1111/j.1399-0012.2009.01048.x

[65] Shakya D K CT. Quality of life of kidney donors residing in Kathmandu valley. J Ren Care. 2016;42:115–122. 26909874 10.1111/jorc.12151

[66] Sharp J McRae A McNeill Y. Decision making and psychosocial outcomes among living kidney donors: a pilot study. Prog Transplant. 2010;20:53–57. 20397347 10.1177/152692481002000109

[67] Shrestha A Shrestha A Vallance C. Quality of life of living kidney donors: a single-center experience. Transplant Proc. 2008;40:1375–1377. 18589110 10.1016/j.transproceed.2008.03.132

[68] Tumin M Abdul Talib Abdul Mutalib M Mohd Satar N. A comparison of donor and control group quality of life. Ann Transplant. 2014;19:112–118. 24584108 10.12659/AOT.889490

[69] Wiedebusch S Reiermann S Steinke C. Quality of life, coping, and mental health status after living kidney donation. Transplant Proc. 2009;41:1483–1488. 19545662 10.1016/j.transproceed.2009.02.102

[70] Zheng XY Han S Wang LM. Quality of life and psychology after living-related kidney transplantation from donors and recipients in China. Transplant Proc. 2014;46:3426–3430. 25498066 10.1016/j.transproceed.2014.04.014

[71] Jaseanchiun W Sirithanaphol W Chotikawanich E. Quality of life after donor nephrectomy for living donor kidney transplantation at Srinagarind Hospital. J Med Assoc Thai. 2012;95(Suppl 11):S15–S17. 23961614

[72] Mjoen G Stavem K Westlie L. Quality of life in kidney donors. Am J Transplant. 2011;11:1315–1319. 21486387 10.1111/j.1600-6143.2011.03517.x

[73] Benzing C Hau HM Kurtz G. Long-term health-related quality of life of living kidney donors: a single-center experience. Qual Life Res. 2015;24:2833–2842. 26149394 10.1007/s11136-015-1027-2

[74] Glotzer OS Singh TP Gallichio MH. Long-term quality of life after living kidney donation. Transplant Proc. 2013;45:3225–3228. 24182789 10.1016/j.transproceed.2013.05.006

[75] Kroencke S Fischer L Nashan B. A prospective study on living related kidney donors’ quality of life in the first year: choosing appropriate reference data. Clin Transplant. 2012;26:E418–E427. 22882697 10.1111/j.1399-0012.2012.01691.x

[76] Meyer KB Hartmann A Mjoen G. Relationships between clinical, self-reported, and donation specific outcomes: a prospective follow-up study 10 years after kidney donation. Ann Transplant. 2017;22:148–155. 28321111 10.12659/AOT.902330 PMC12577502

[77] Keys DO Jackson S Berglund D. Kidney donor outcomes ≥ 50 years after donation. Clin Transplant. 2019;33:e13657. 31283043 10.1111/ctr.13657

[78] Klop KW Dols LF Weimar W. Quality of life of elderly live kidney donors. Transplantation. 2013;96:644–648. 23860088 10.1097/TP.0b013e31829e6d9b

[79] Minnee RC Bemelman WA Polle SW. Older living kidney donors: surgical outcome and quality of life. Transplantation. 2008;86:251–256. 18645487 10.1097/TP.0b013e31817789dd

[80] O’Keeffe LM Ramond A Oliver-Williams C. Mid- and long-term health risks in living kidney donors: a systematic review and meta-analysis. Ann Intern Med. 2018;168:276–284. 29379948 10.7326/M17-1235

[81] Ku JH. Health-related quality of life of living kidney donors: review of the short form 36-health questionnaire survey. Transpl Int. 2005;18:1309–1317. 16297049 10.1111/j.1432-2277.2005.00231.x

[82] Liu S Zhou X Dai H. Assessing health-related quality of life of living kidney donors using the 36-item medical outcomes Short-Form-36 questionnaire: a meta-analysis. Psychol Health Med. 2021;26:917–930. 33332156 10.1080/13548506.2020.1856392

[83] Morgan BR Ibrahim HN. Long-term outcomes of kidney donors. Arab J Urol. 2011;9:79–84. 26579273 10.1016/j.aju.2011.06.006 PMC4150560

[84] Reese PP Boudville N Garg AX. Living kidney donation: outcomes, ethics, and uncertainty. Lancet. 2015;385:2003–2013. 26090646 10.1016/S0140-6736(14)62484-3

[85] Slinin Y Brasure M Eidman K. Long-term outcomes of living kidney donation. Transplantation. 2016;100:1371–1386. 29543690 10.1097/TP.0000000000001252

[86] Soneji ND Vyas J Papalois VE. Long-term donor outcomes after living kidney donation. Exp Clin Transplant. 2008;6:215–223. 18954300

[87] Albertsmeyer S Renner FC Yildiz S. One hundred six live kidney donors in a single German transplantation center: renal, physical, and psychological follow-up. Transplant Proc. 2010;42:3992–3993. 21168608 10.1016/j.transproceed.2010.09.039

[88] Tellioglu G Berber I Yatkin I. Quality of life analysis of renal donors. Transplant Proc. 2008;40:50–52. 18261545 10.1016/j.transproceed.2007.11.030

[89] Zargooshi J. Quality of life of Iranian kidney “donors”. J Urol. 2001;166:1790–1799. 11586226

[90] Fallahzadeh MK Jafari L Roozbeh J. Comparison of health status and quality of life of related versus paid unrelated living kidney donors. Am J Transplant. 2013;13:3210–3214. 24266971 10.1111/ajt.12488

[91] Fry-Revere S Chen D Bastani B. Coercion, dissatisfaction, and social stigma: an ethnographic study of compensated living kidney donation in Iran. Int Urol Nephrol. 2020;52:2403–2414. 29480443 10.1007/s11255-018-1824-y

[92] Schnitzbauer AA Hornung M Seidel U. Does mini-incision donor nephrectomy improve quality of life in living kidney donors? Clin Transplant. 2007;21:235–240. 17425751 10.1111/j.1399-0012.2006.00631.x

[93] Giessing M Reuter S Deger S. Laparoscopic versus open donor nephrectomy in Germany: impact on donor health-related quality of life and willingness to donate. Transplant Proc. 2005;37:2011–2015. 15964326 10.1016/j.transproceed.2005.03.007

[94] Rodrigue JR Cross NJ Newman RC. Patient-reported outcomes for open versus laparoscopic living donor nephrectomy. Prog Transplant. 2006;16:162–169. 16789708 10.1177/152692480601600213

[95] Vernadakis S Marinaki S Darema M. The evolution of living donor nephrectomy program at a hellenic transplant center. laparoscopic vs. open donor nephrectomy: single-center experience. J Clin Med. 2021;10:1195. 33809339 10.3390/jcm10061195 PMC8001196

[96] Andersen MH Mathisen L Veenstra M. Quality of life after randomization to laparoscopic versus open living donor nephrectomy: long-term follow-up. Transplantation. 2007;84:64–69. 17627239 10.1097/01.tp.0000268071.63977.42

[97] Buell JF Lee L Martin JE. Laparoscopic donor nephrectomy vs. open live donor nephrectomy: a quality of life and functional study. Clin Transplant. 2005;19:102–109. 15659142 10.1111/j.1399-0012.2004.00308.x

[98] Dahm F Weber M Muller B. Open and laparoscopic living donor nephrectomy in Switzerland: a retrospective assessment of clinical outcomes and the motivation to donate. Nephrol Dial Transplant. 2006;21:2563–2568. 16702206 10.1093/ndt/gfl207

[99] Friedersdorff F Kothmann L Manus P. Long-term donor outcomes after pure laparoscopic versus open living donor nephrectomy: focus on pregnancy rates, hypertension and quality of life. Urol Int. 2016;97:450–456. 27577572 10.1159/000447064

[100] Kok NF Alwayn IP Tran KT. Psychosocial and physical impairment after mini-incision open and laparoscopic donor nephrectomy: a prospective study. Transplantation. 2006;82:1291–1297. 17130777 10.1097/01.tp.0000239312.45050.05

[101] Han WK Lee HY Jeon HG. Quality of life comparison between open and retroperitoneal video-assisted minilaparotomy surgery for kidney donors. Transplant Proc. 2010;42:1479–1483. 20620458 10.1016/j.transproceed.2010.01.070

[102] Hoda MR Hamza A Wagner S. Impact of hand-assisted laparoscopic living donor nephrectomy on donor’s quality of life, emotional, and social state. Transplant Proc. 2010;42:1487–1491. 20620460 10.1016/j.transproceed.2010.01.078

[103] Wahba R Vitinius F Walczuch B. Hand-assisted retroperitoneoscopic donor nephrectomy compared to anterior approach open donor nephrectomy: improved long-term physical component score in health-related quality of life in living kidney donors. Transplant Proc. 2021;53:786–792. 32981693 10.1016/j.transproceed.2020.08.026

[104] Garcia-Ochoa C Feldman LS Nguan C. Impact of perioperative complications on living kidney donor health-related quality of life and mental health: results from a prospective cohort study. Can J Kidney Health Dis. 2021;8:20543581211037429. 34394947 10.1177/20543581211037429 PMC8361543

[105] Ozcurumez G Tanriverdi N Colak T. The psychosocial impact of renal transplantation on living related donors and recipients: preliminary report. Transplant Proc. 2004;36:114–116. 15013317 10.1016/j.transproceed.2003.11.004

[106] Pocnet C Antonietti JP Strippoli MF. Individuals’ quality of life linked to major life events, perceived social support, and personality traits. Qual Life Res. 2016;25:2897–2908. 27098234 10.1007/s11136-016-1296-4

[107] Cleland C Kearns A Tannahill C. The impact of life events on adult physical and mental health and well-being: longitudinal analysis using the GoWell health and well-being survey. BMC Res Notes. 2016;9:470. 27760568 10.1186/s13104-016-2278-x PMC5070029

[108] Han X Lim JY Raman L. Nephrectomy-induced reduced renal function and the health-related quality of life of living kidney donors. Clin Transplant. 2017;31:e12910. 10.1111/ctr.12910 28083977

[109] Maple H Chilcot J Burnapp L. Motivations, outcomes, and characteristics of unspecified (nondirected altruistic) kidney donors in the United Kingdom. Transplantation. 2014;98:1182–1189. 25099701 10.1097/TP.0000000000000340

[110] Rodrigue JR Schutzer ME Paek M. Altruistic kidney donation to a stranger: psychosocial and functional outcomes at two US transplant centers. Transplantation. 2011;91:772–778. 21285916 10.1097/TP.0b013e31820dd2bd

[111] Serur D Bretzlaff G Christos P. Solicited kidney donors: are they coerced? Nephrology. 2015;20:952–955. 26511772 10.1111/nep.12551 PMC4756477

[112] Pollmann I Gueler F Mikuteit M. Adaptive personality traits and psychosocial correlates among living kidney donors. Front Psychiatry. 2017;8:210. 29109691 10.3389/fpsyt.2017.00210 PMC5660284

[113] Owen M Lorgelly P Serpell M. Chronic pain following donor nephrectomy—a study of the incidence, nature and impact of chronic post-nephrectomy pain. Eur J Pain. 2010;14:732–734. 20064733 10.1016/j.ejpain.2009.11.013

[114] Slakey DP Altman AM Bhutto J. Psychosocial aspects of laparoscopic donor nephrectomy: donor impressions. Dial Transplant. 2008;37:160–165.

[115] Yasumura T Nakai I Oka T. Experience with 247 living related donor nephrectomy cases at a single institution in Japan. Jpn J Surg. 1988;18:252–258. 3043068 10.1007/BF02471441

[116] Thys K Schwering KL Siebelink M; ELPAT Pediatric Organ Donation and Transplantation Working Group. Psychosocial impact of pediatric living-donor kidney and liver transplantation on recipients, donors, and the family: a systematic review. Transpl Int. 2015;28:270–280. 25363518 10.1111/tri.12481

[117] Roberts LA West-Wooding U. The living kidney donor perspective in a transplant programme in Trinidad and Tobago: seeing donation through the donor’s eyes. West Indian Med J. 2017;66:697.

[118] Katvan E Cohen J Rahamimov R. A Comparison of recalled pain memory following living kidney donation between directed and non-directed, altruistic donors. Prog Transplant. 2022;32:285–291. 36039525 10.1177/15269248221122897

[119] Sharma VK Enoch MD. Psychological sequelae of kidney donation. A 5-10 year follow up study. Acta Psychiatr Scand. 1987;75:264–267. 3296664 10.1111/j.1600-0447.1987.tb02787.x

[120] Jordan J Sann U Janton A. Living kidney donors’ long-term psychological status and health behavior after nephrectomy—a retrospective study. J Nephrol. 2004;17:728–735. 15593042

[121] Timmerman L Laging M Westerhof GJ. Mental health among living kidney donors: a prospective comparison with matched controls from the general population. Am J Transplant. 2015;15:508–517. 25582231 10.1111/ajt.13046

[122] Tanriverdi N Ozcurumez G Colak T. Quality of life and mood in renal transplantation recipients, donors, and controls: preliminary report. Transplant Proc. 2004;36:117–119. 15013318 10.1016/j.transproceed.2003.11.003

[123] Taskintuna N Ozcurumez G Duru C. Psychosocial aspects of living-related donor renal transplantation: quality of life and mood in recipients, donors and controls. Int J Psychiatry Clin Pract. 2009;13:218–222. 24916823 10.1080/13651500902846447

[124] Oguten EG Barlas IS Akin EB. Mental distress symptoms and life satisfaction among living kidney donors: frequency and association with subjective evaluations. Transplant Proc. 2019;51:2232–2236. 31399201 10.1016/j.transproceed.2019.01.154

[125] Frade IC Fonseca I Dias L. Impact assessment in living kidney donation: psychosocial aspects in the donor. Transplant Proc. 2008;40:677–681. 18454984 10.1016/j.transproceed.2008.02.036

[126] Lopes A Frade IC Teixeira L. Depression and anxiety in living kidney donation: evaluation of donors and recipients. Transplant Proc. 2011;43:131–136. 21335170 10.1016/j.transproceed.2010.12.028

[127] Karagöl A. Levels of depression, anxiety and quality-of-life of kidney and liver donors in a university hospital in Ankara. Anadolu Psikiyatr Derg. 2019;20:175–181.

[128] Chen P Luo Q Peng L. Anxiety and decreased social support underline poorer quality of life of parent living kidney donors. Asia Pac Psychiatry. 2015;7:197–205. 23857596 10.1111/appy.12087

[129] Karagol A Torenli Kaya Z. Evaluating early maladaptive schemas and depression levels in living kidney and liver donors. Psychol Health Med. 2022;27:2161–2170. 34545761 10.1080/13548506.2021.1981409

[130] Chen CH Chen Y Chiang YJ. Risks and quality-of-life changes in living kidney donors. Transplant Proc. 2004;36:1920–1921. 15518699 10.1016/j.transproceed.2004.08.016

[131] Haljamae U Nyberg G Sjostrom B. Remaining experiences of living kidney donors more than 3 yr after early recipient graft loss. Clin Transplant. 2003;17:503–510. 14756265 10.1046/j.1399-0012.2003.00078.x

[132] Woldemichael A Berhanu E Fritsch C. Psychological well-being of living kidney donors and recipients. Exp Clin Transplant. 2021;19:779–787. 34269647 10.6002/ect.2020.0423

[133] Massey EK Kranenburg LW Zuidema WC. Encouraging psychological outcomes after altruistic donation to a stranger. Am J Transplant. 2010;10:1445–1452. 20486913 10.1111/j.1600-6143.2010.03115.x

[134] Timmerman L Zuidema WC Erdman RA. Psychologic functioning of unspecified anonymous living kidney donors before and after donation. Transplantation. 2013;95:1369–1374. 23542471 10.1097/TP.0b013e31828eaf81

[135] Massey EK Pronk MC Zuidema WC. Positive and negative aspects of mental health after unspecified living kidney donation: a cohort study. Br J Health Psychol. 2022;27:374–389. 34296497 10.1111/bjhp.12549 PMC9291094

[136] Wadström J von Zur-Mühlen B Lennerling A. Living anonymous renal donors do not regret: intermediate and long-term follow-up with a focus on motives and psychosocial outcomes. Ann Transplant. 2019;24:234–241. 31023996 10.12659/AOT.913827 PMC6507493

[137] Jacobs C Berglund DM Wiseman JF. Long-term psychosocial outcomes after nondirected donation: a single-center experience. Am J Transplant. 2019;19:1498–1506. 30417522 10.1111/ajt.15179

[138] Hamama-Raz Y Ring L Mahat-Shamir M. Death anxiety and psychological distress post-donation in non-directed living kidney donors. Death Stud. 2020;44:490–497. 30907706 10.1080/07481187.2019.1586793

[139] Ehlers M Vitinius F Langenbach M. Altruistic nondirected kidney donation: attitudes, characteristics and ethical implications. Curr Opin Organ Transplant. 2017;22:584–587. 28857843 10.1097/MOT.0000000000000462

[140] Pronk MC Zuidema WC Weimar W. Twenty years of unspecified kidney donation: unspecified donors looking back on their donation experiences. Transpl Int. 2023;36:10959. 36925946 10.3389/ti.2023.10959 PMC10011065

[141] Tong A Craig JC Wong G. “It was just an unconditional gift.” Self reflections of non-directed living kidney donors. Clin Transplant. 2012;26:589–599. 22251271 10.1111/j.1399-0012.2011.01578.x

[142] Clarke A Mitchell A Abraham C. Understanding donation experiences of unspecified (altruistic) kidney donors. Br J Health Psychol. 2014;19:393–408. 23692296 10.1111/bjhp.12048

[143] Lim WH Chan KE Ng CH. A qualitative systematic review of anonymous/unspecified living kidney and liver donors’ perspectives. PLoS One. 2022;17:e0277792. 36584032 10.1371/journal.pone.0277792 PMC9803135

[144] Clemens K Boudville N Dew MA; Donor Nephrectomy Outcomes Research (DONOR) Network. The long-term quality of life of living kidney donors: a multicenter cohort study. Am J Transplant. 2011;11:463–469. 21342446 10.1111/j.1600-6143.2010.03424.x

[145] Manera KE Hanson CS Chapman JR. Expectations and experiences of follow-up and self-care after living kidney donation: a focus group study. Transplantation. 2017;101:2627–2635. 28538499 10.1097/TP.0000000000001771

[146] Leichtman A Abecassis M Barr M; Living Kidney Donor Follow-Up Conference Writing Group. Living kidney donor follow-up: state-of-the-art and future directions, conference summary and recommendations. Am J Transplant. 2011;11:2561–2568. 22054039 10.1111/j.1600-6143.2011.03816.x

[147] Tong A Chapman JR Wong G. The motivations and experiences of living kidney donors: a thematic synthesis. Am J Kidney Dis. 2012;60:15–26. 22305757 10.1053/j.ajkd.2011.11.043

[148] Ummel D Achille M Mekkelholt J. Donors and recipients of living kidney donation: a qualitative metasummary of their experiences. J Transplant. 2011;2011:626501. 21766008 10.1155/2011/626501 PMC3134215

[149] Brown JB Karley ML Boudville N. The experience of living kidney donors. Health Soc Work. 2008;33:93–100. 18510123 10.1093/hsw/33.2.93

[150] Frade IC Lopes A Teixeira L. Perceptions in living kidney donation: what protagonists think and feel. Transplant Proc. 2011;43:39–42. 21335149 10.1016/j.transproceed.2010.12.029

[151] Heck G Schweitzer J Seidel-Wiesel M. Psychological effects of living related kidney transplantation - risks and chances. Clin Transplant. 2004;18:716–721. 15516249 10.1111/j.1399-0012.2004.00285.x

[152] Kemph JP Bermann EA Coppolillo HP. Kidney transplant and shifts in family dynamics. Am J Psychiatry. 1969;125:1485–1490. 4887670 10.1176/ajp.125.11.1485

[153] Langenbach M Stippel A Stippel D. Kidney donors’ quality of life and subjective evaluation at 2 years after donation. Transplant Proc. 2009;41:2512–2514. 19715964 10.1016/j.transproceed.2009.06.122

[154] Pradel FG Mullins CD Bartlett ST. Exploring donors’ and recipients’ attitudes about living donor kidney transplantation. Prog Transplant. 2003;13:203–210. 14558635 10.1177/152692480301300307

[155] Williams AM Colefax L O’Driscoll CT. An exploration of experiences of living renal donors following donation. Nephrol Nurs J. 2009;36:423–427. 19715110

[156] Lunsford SL Shilling LM Chavin KD. Racial differences in the living kidney donation experience and implications for education. Prog Transplant. 2007;17:234–240. 17944164 10.1177/152692480701700312

[157] Yucetin L Bozoklar CA Yanik O. An investigation of post-traumatic growth experiences among living kidney donors. Transplant Proc. 2015;47:1287–1290. 26093699 10.1016/j.transproceed.2015.04.027

[158] Achille M Soos J Fortin MC. Differences in psychosocial profiles between men and women living kidney donors. Clin Transplant. 2007;21:314–320. 17488379 10.1111/j.1399-0012.2007.00641.x

[159] Pronk MC Zuidema W Weimar W. Reflections of unspecified anonymous kidney donors on their motivation and the impact of donation on their mental health: a qualitative study. SSM. 2023;3:100272.

[160] Kowal K Zatorski M Kwiatkowski A. Experiencing one’s own body and body image in living kidney donors—a sociological and psychological study. PLoS One. 2021;16:e0249397. 33857150 10.1371/journal.pone.0249397 PMC8049271

[161] Joshi SA Almeida N Almeida A. Assessment of the perceived quality of life of successful kidney transplant recipients and their donors pre- and post-transplantation. Transplant Proc. 2013;45:1435–1437. 23726590 10.1016/j.transproceed.2013.01.037

[162] Ralph AF Butow P Craig JC. Living kidney donor and recipient perspectives on their relationship: longitudinal semi-structured interviews. BMJ Open. 2019;9:e026629. 10.1136/bmjopen-2018-026629 PMC6500358 30948607

[163] Ralph AF Butow P Hanson CS. Donor and recipient views on their relationship in living kidney donation: thematic synthesis of qualitative studies. Am J Kidney Dis. 2017;69:602–616. 27889296 10.1053/j.ajkd.2016.09.017

[164] Ummel D Achille M. Transplant trajectory and relational experience within living kidney dyads. Qual Health Res. 2016;26:194–203. 25700284 10.1177/1049732315570128

[165] Vlaovic PD Devins GM Abbey S. Psychosocial impact of renal donation. Can J Urol. 1999;6:859–864. 11180783

[166] Baines LS Beattie TJ Murphy AV. Relationship between donors and pediatric recipients of kidney transplants: a psychosocial study. Transplant Proc. 2001;33:1897–1899. 11267560 10.1016/s0041-1345(00)02742-1

[167] Farahani ZB Esmaeili M Salsali M. Living related transplantation: the outcomes of kidney donation in Iran. Acta Med Mediterr. 2016;32:1071–1076.

[168] Neuhaus TJ Wartmann M Weber M. Psychosocial impact of living-related kidney transplantation on donors and partners. Pediatr Nephrol. 2005;20:205–209. 15627165 10.1007/s00467-004-1749-9

[169] Van Pilsum Rasmussen SE Robin M Saha A. The tangible benefits of living donation: results of a qualitative study of living kidney donors. Transplant Direct. 2020;6:e626. 33204824 10.1097/TXD.0000000000001068 PMC7665258

[170] Nohre M Pollmann I Mikuteit M. Partnership satisfaction in living kidney donors. Front Psychiatr. 2018;9:353. 10.3389/fpsyt.2018.00353 PMC6085414 30123146

[171] Serur D Charlton M Bretzlaff G. Is donating a kidney to a friend bad for your marriage? Nephrology. 2015;20:434–436. 25900385 10.1111/nep.12426

[172] Serur D Charlton M Lawton M. Donors in chains: psychosocial outcomes of kidney donors in paired exchange. Prog Transplant. 2014;24:371–374. 25488561 10.7182/pit2014222

[173] Al Breizat AH Abunaser MT Al Breizat Z. Living donors: altruism and feeling forgotten. Exp Clin Transplant. 2020;18(Suppl 1):22–28. 32008488 10.6002/ect.TOND-TDTD2019.L25

[174] Clarke A Mitchell A Abraham C. Understanding donation experiences of unspecified (altruistic) kidney donors. Br J Health Psychol. 2014;19:393–408. 23692296 10.1111/bjhp.12048

[175] Meyer KB Bjork IT Wahl AK. Long-term experiences of Norwegian live kidney donors: qualitative in-depth interviews. BMJ Open. 2017;7:e014072. 10.1136/bmjopen-2016-014072 PMC5318577 28209606

[176] Greif-Higer G Wandel E Otto G. Psychological conflicts between relatives during the long-term course after successful living organ donation. Transplant Proc. 2008;40:902–906. 18555075 10.1016/j.transproceed.2008.03.040

[177] Kisch AM Forsberg A Fridh I. The meaning of being a living kidney, liver, or stem cell donor-a meta-ethnography. Transplantation. 2018;102:744–756. 29298236 10.1097/TP.0000000000002073

[178] Ho EW Murillo AL Davis LA. Findings of living donation experiences shared on a digital storytelling platform: a thematic analysis. PEC Innov. 2022;1:100023. 37213721 10.1016/j.pecinn.2022.100023 PMC10194229

[179] Davis L Iraheta YA Ho EW. Living kidney donation stories and advice shared through a digital storytelling library: a qualitative thematic analysis. Kidney Med. 2022;4:100486. 35755303 10.1016/j.xkme.2022.100486 PMC9218227

[180] Fehrman-Ekholm I Brink B Ericsson C. Kidney donors don’t regret: follow-up of 370 donors in Stockholm since 1964. Transplantation. 2000;69:2067–2071. 10852598 10.1097/00007890-200005270-00016

[181] Rana TA Akoh JA. Donor perspectives in living kidney transplantation. Dial Transplant. 2010;39:208–213.

[182] Sahay M Anuradha NG. Risk of live kidney donation—Indian perspective. J Assoc Physicians India. 2007;55:267–270. 17694785

[183] Weitz J Koch M Mehrabi A. Living-donor kidney transplantation: risks of the donor—benefits of the recipient. Clin Transplant. 2006;20(Suppl 17):13–16. 17100696 10.1111/j.1399-0012.2006.00595.x

[184] Binet I Bock AH Vogelbach P. Outcome in emotionally related living kidney donor transplantation. Nephrol Dial Transplant. 1997;12:1940–1948. 9306347 10.1093/ndt/12.9.1940

[185] Heidary Rouchi A Mahdavi-Mazdeh M Zamyadi M. Compensated living kidney donation in Iran: donor’s attitude and short-term follow-up. Iran J Kidney Dis. 2009;3:34–39. 19377257

[186] Jawad F Zafar MN Aziz T. Living kidney donation-benefits of a follow up clinic. Transplant Proc. 2003;35:2561. 14612016 10.1016/j.transproceed.2003.09.077

[187] Oliveira B Mascarenhas C Cardoso G. Assessment of the degree of satisfaction among living kidney donors. Transplant Proc. 2011;43:43–47. 21335150 10.1016/j.transproceed.2010.12.043

[188] Bieniasz M Kieszek R Jakubowska-Winecka A. Psychological aspects of living kidney donation in Poland: experience of one center. Transplant Proc. 2018;50:1637–1639. 30056874 10.1016/j.transproceed.2018.04.054

[189] Burroughs TE Waterman AD Hong BA. One organ donation, three perspectives: experiences of donors, recipients, and third parties with living kidney donation. Prog Transplant. 2003;13:142–150. 12841522 10.1177/152692480301300212

[190] Corley MC Elswick RK Sargeant CC. Attitude, self-image, and quality of life of living kidney donors. Nephrol Nurs J. 2000;27:43–50; discussion 51. 10852690

[191] Skaczkowski G Barrett A Olver I. ‘It is a life changing experience’: the experiences of living kidney donors who live in rural Australia. Aust J Rural Health. 2023;31:866–877. 37335838 10.1111/ajr.13008

[192] Kaul A Bhaduaria D Behera MR. Psycho-social health and quality of life among kidney donors following transplantation. Transpl Immunol. 2022;74:101649. 35777614 10.1016/j.trim.2022.101649

[193] Feltrin A Pegoraro R Rago C. Experience of donation and quality of life in living kidney and liver donors. Transpl Int. 2008;21:466–472. 18225994 10.1111/j.1432-2277.2007.00632.x

[194] Walsh A. Living kidney donor experiences: implications for counselling. EDTNA ERCA J. 2004;30:196–200. 15835410 10.1111/j.1755-6686.2004.tb00367.x

[195] Hildebrand L Melchert TP Anderson RC. Impression management during evaluation and psychological reactions post-donation of living kidney donors. Clin Transplant. 2014;28:855–861. 24888484 10.1111/ctr.12390

[196] Garcia MF Andrade LG Carvalho MF. Living kidney donors—prospective study of quality of life before and after kidney donation. Clin Transplant. 2013;27:9–14. 10.1111/j.1399-0012.2012.01687.x 22831164

[197] Li T Dokus MK Kelly KN. Survey of living organ donors’ experience and directions for process improvement. Prog Transplant. 2017;27:232–239. 29187096 10.1177/1526924817715467

[198] Brown JB Karley ML Boudville N. Living kidney donors’ experiences with the health care system. Soc Work Health Care. 2008;46:53–68. 18551829 10.1300/J010v46n03_03

[199] Shaw RM Bell LJ. ‘Because you can’t live on love’: living kidney donors’ perspectives on compensation and payment for organ donation. Health Expect. 2015;18:3201–3212. 25418552 10.1111/hex.12310 PMC5810734

[200] Wiseman JF Jacobs CL Larson DB. Financial burden borne by laparoscopic living kidney donors. Transplantation. 2017;101:2253–2257. 27941440 10.1097/TP.0000000000001568

[201] Barnieh L Arnold JB Boudville N; Donor Nephrectomy Outcomes Research (DONOR) Network. Living kidney donors’ financial expenses and mental health. Transplantation. 2021;105:1356–1364. 33741846 10.1097/TP.0000000000003401

[202] Wolters HH Heidenreich S Senninger N. Living donor kidney transplantation: chance for the recipient--financial risk for the donor? Transplant Proc. 2003;35:2091–2092. 14529850 10.1016/s0041-1345(03)00675-4

[203] Maghen A Mendoza G Vargas GB. How can we help alleviate the financial concerns of non-directed (altruistic) living kidney donors? Prog Transplant. 2021;31:19–26. 33292055 10.1177/1526924820978589

[204] Klarenbach S Gill JS Knoll G; Donor Nephrectomy Outcomes Research (DONOR) Network. Economic consequences incurred by living kidney donors: a Canadian multi-center prospective study. Am J Transplant. 2014;14:916–922. 24597854 10.1111/ajt.12662 PMC4285205

[205] Rodrigue JR Schold JD Morrissey P; KDOC Study Group. Direct and indirect costs following living kidney donation: findings from the KDOC study. Am J Transplant. 2016;16:869–876. 26845630 10.1111/ajt.13591

[206] Clarke KS Klarenbach S Vlaicu S; Donor Nephrectomy Outcomes Research (DONOR) Network. The direct and indirect economic costs incurred by living kidney donors—a systematic review. Nephrol Dial Transplant. 2006;21:1952–1960. 16554329 10.1093/ndt/gfl069

[207] Fu R Sekercioglu N Hishida M. Economic consequences of adult living kidney donation: a systematic review. Value Health. 2021;24:592–601. 33840438 10.1016/j.jval.2020.10.005

[208] Rodrigue JR Schold JD Mandelbrot DA. Concern for lost income following donation deters some patients from talking to potential living donors. Prog Transplant. 2016;26:292–298. 27495327 10.1177/1526924816661332

[209] Park S Park J Kang E. Economic impact of donating a kidney on living donors: a Korean cohort study. Am J Kidney Dis. 2022;79:175–184.e1. 34419516 10.1053/j.ajkd.2021.07.009

[210] Garg N Waterman AD Ranasinghe O. Wages, travel, and lodging reimbursement by the National Kidney Registry: an important step toward financial neutrality for living kidney donors in the United States. Transplantation. 2021;105:2606–2611. 33675322 10.1097/TP.0000000000003721

[211] Frech A Natale G Hayes D. Marital status and return to work after living kidney donation. Prog Transplant. 2018;28:226–230. 29879858 10.1177/1526924818781560

[212] Tushla L Rudow DL Milton J; American Society of Transplantation. Living-donor kidney transplantation: reducing financial barriers to live kidney donation—recommendations from a consensus conference. Clin J Am Soc Nephrol. 2015;10:1696–1702. 26002904 10.2215/CJN.01000115 PMC4559503

[213] Salomon DR Langnas AN Reed AI; AST/ASTS Incentives Workshop Group (IWG). AST/ASTS workshop on increasing organ donation in the United States: creating an “arc of change” from removing disincentives to testing incentives. Am J Transplant. 2015;15:1173–1179. 25833653 10.1111/ajt.13233

[214] Hays R Rodrigue JR Cohen D. Financial neutrality for living organ donors: reasoning, rationale, definitions, and implementation strategies. Am J Transplant. 2016;16:1973–1981. 27037542 10.1111/ajt.13813

[215] Gill JS Delmonico F Klarenbach S. Providing coverage for the unique lifelong health care needs of living kidney donors within the framework of financial neutrality. Am J Transplant. 2017;17:1176–1181. 27888569 10.1111/ajt.14147

[216] Davis CL Cooper M. The state of U.S. living kidney donors. Clin J Am Soc Nephrol. 2010;5:1873–1880. 20634322 10.2215/CJN.01510210 PMC2974389

[217] Gibney EM Doshi MD Hartmann EL. Health insurance status of US living kidney donors. Clin J Am Soc Nephrol. 2010;5:912–916. 20413444 10.2215/CJN.07121009 PMC2863970

[218] Rodrigue JR Fleishman A. Health insurance trends in United States living kidney donors (2004 to 2015). Am J Transplant. 2016;16:3504–3511. 27088263 10.1111/ajt.13827 PMC5069113

[219] Boyarsky BJ Massie AB Alejo JL. Experiences obtaining insurance after live kidney donation. Am J Transplant. 2014;14:2168–2172. 25041695 10.1111/ajt.12819 PMC4194161

[220] Dew MA Myaskovsky L Steel JL. Managing the psychosocial and financial consequences of living donation. Curr Transplant Rep. 2014;1:24–34. 24592353 10.1007/s40472-013-0003-4 PMC3938191

[221] Yang RC Thiessen-Philbrook H Klarenbach S; Donor Nephrectomy Outcomes Research (DONOR) Network. Insurability of living organ donors: a systematic review. Am J Transplant. 2007;7:1542–1551. 17430400 10.1111/j.1600-6143.2007.01793.x

[222] Wu DA Robb ML Watson CJE. Barriers to living donor kidney transplantation in the United Kingdom: a national observational study. Nephrol Dial Transplant. 2017;32:890–900. 28379431 10.1093/ndt/gfx036 PMC5427518

[223] Taler SJ Messersmith EE Leichtman AB; RELIVE Study Group. Demographic, metabolic, and blood pressure characteristics of living kidney donors spanning five decades. Am J Transplant. 2013;13:390–398. 23137211 10.1111/j.1600-6143.2012.04321.x PMC3558745

[224] Schold JD Goldfarb DA Buccini LD. Comorbidity burden and perioperative complications for living kidney donors in the United States. Clin J Am Soc Nephrol. 2013;8:1773–1782. 24071651 10.2215/CJN.12311212 PMC3789355

[225] Ahmadi AR Lafranca JA Claessens LA. Shifting paradigms in eligibility criteria for live kidney donation: a systematic review. Review. 2015;87:31–45. 10.1038/ki.2014.118 24786706

[226] Wirken L van Middendorp H Hooghof CW. Development and feasibility of a guided and tailored internet-based cognitive-behavioural intervention for kidney donors and kidney donor candidates. BMJ Open. 2018;8:e020906. 10.1136/bmjopen-2017-020906 PMC6042571 29961018

[227] Dew MA DiMartini AF DeVito Dabbs AJ. Preventive intervention for living donor psychosocial outcomes: feasibility and efficacy in a randomized controlled trial. Am J Transplant. 2013;13:2672–2684. 23924065 10.1111/ajt.12393 PMC3837427

[228] Mamode N Bestard O Claas F. European guideline for the management of kidney transplant patients with HLA antibodies: by the European Society for Organ Transplantation Working Group. Transpl Int. 2022;35:10511. 36033645 10.3389/ti.2022.10511 PMC9399356

