# Supplementary material for: Living Kidney Donation: A Narrative Review of Mid- and Long-term Psychosocial Outcomes
Source: Transplantation. 2024 Jun 18;109(2):259–72. doi: 10.1097/TP.0000000000005094 (PMC11652709; doi:10.1097/TP.0000000000005094)
Supplement: Supplementary file 1 [file tpa-109-0259-s001.pdf]

Supplementary Material

Appendix A: Search string per database

Medline

((*"Living Donors"/ AND (exp *"Kidney"/ OR *"Kidney Transplantation"/)) OR (((kidney* OR renal* OR nephrectom*) ADJ3 (donor* OR donat*)) OR ((living OR live OR altruist*) ADJ3 (donor* OR donat*)) OR ((living OR live OR altruist*) ADJ3 (kidney*))).ti.) AND (exp *"Psychology"/ OR exp *"Mental Health"/ OR exp *"Depression"/ OR exp *"Anxiety"/ OR *"Euphoria"/ OR *"Happiness"/ OR (psychol* OR mental-health* OR well-being* OR wellbeing* OR (stress* NOT oxidative-stress*) OR cost* OR economic* OR financial* OR coverage* OR insur* OR emotion* OR personal-benef* OR perceived-benef* OR coping OR ((qualit*) ADJ3 (life)) OR qol OR depress* OR anxiety OR anxious OR euphor* OR happiness* OR happy OR satisfact* OR dissatisfact* OR regret* OR feel* OR ((patient* OR donor* OR donat* OR individual* OR living OR live) ADJ3 (experien* OR outcome* OR percept* OR attitude* OR perspect*)) OR psychosocial* OR psycho-social* OR ((chronic* OR post-operat* OR postoperat* OR post-donation* OR postdonation*) ADJ3 (pain)) OR benefit* OR ineligib* OR health-risk* OR health-utilit* OR expectation* OR motivation* OR self-reflect* OR mood).ti,kf.) NOT (news OR congres* OR abstract* OR book* OR chapter* OR dissertation abstract*).pt. NOT (exp animals/ NOT humans/) NOT (exp *"Transplantation, Heterologous"/ OR (xenotransplant*).ti.) AND (english).lg **NOT** (liver* NOT (kidney* OR nephr*)).ti.

Embase

(('living donor'/exp/mj AND ('kidney'/exp/mj OR 'kidney transplantation'/exp/mj)) OR (((kidney* OR renal* OR nephrectom*) NEAR/3 (donor* OR donat*)) OR ((living OR live OR altruist*) NEAR/3 (donor* OR donat*)) OR ((living OR live OR altruist*) NEAR/3 (kidney*))):ti) **AND** ('psychology'/exp/mj OR 'mental health'/exp/mj OR 'depression'/exp/mj OR 'anxiety'/exp/mj OR 'euphoria'/exp/mj OR 'happiness'/exp/mj OR (psychol* OR mental-health* OR well-being* OR wellbeing* OR (stress* NOT oxidative-stress*) OR cost* OR economic* OR financial* OR coverage* OR insur* OR emotion* OR personal-benef* OR perceived-benef* OR coping OR ((qualit*) NEAR/3 (life)) OR qol OR depress* OR anxiety OR anxious OR euphor* OR happiness* OR happy OR satisfact* OR dissatisfact* OR regret* OR feel* OR ((patient* OR donor* OR donat* OR individual* OR living OR live) NEAR/3 (experien* OR outcome* OR percept* OR attitude* OR perspect*)) OR psychosocial* OR psycho-social* OR ((chronic* OR post-operat* OR postoperat* OR post-donation* OR postdonation*) NEAR/3 (pain)) OR benefit* OR ineligib* OR health-risk* OR health-utilit* OR expectation* OR motivation* OR self-reflect* OR mood):ti) **NOT** ([Conference Abstract]/lim OR [preprint]/lim) **NOT** ((animal/exp OR animal*:de OR nonhuman/de) NOT ('human'/exp)) **NOT** ('xenotransplantation'/exp/mj OR (xenotransplant*):ti) **AND** [ENGLISH]/lim **NOT** (liver* NOT (kidney* OR nephr*)):ti

PsycINFO

((((kidney* OR renal* OR nephrectom*) ADJ3 (donor* OR donat*)) OR ((living OR live OR altruist*) ADJ3 (donor* OR donat*)) OR ((living OR live OR altruist*) ADJ3 (kidney*))).ti.) **AND** (exp *psychology/ OR *mental health/ OR *"depression (emotion)"/ OR exp *anxiety/ OR *euphoria/ OR *happiness/ OR (psychol* OR mental-health* OR well-being* OR wellbeing* OR (stress* NOT oxidative-stress*) OR cost* OR economic* OR financial* OR coverage* OR insur* OR emotion* OR personal-benef* OR perceived-benef* OR coping OR ((qualit*) ADJ3 (life)) OR qol OR depress* OR anxiety OR anxious OR euphor* OR happiness* OR happy OR satisfact* OR dissatisfact* OR regret* OR feel* OR ((patient* OR donor* OR donat* OR individual* OR living OR live) ADJ3 (experien* OR outcome* OR percept* OR attitude* OR perspect*)) OR psychosocial* OR psycho-social* OR ((chronic* OR post-operat* OR postoperat* OR post-donation* OR postdonation*) ADJ3 (pain)) OR benefit* OR ineligib* OR health-risk* OR health-utilit* OR expectation* OR motivation* OR self-reflect* OR mood).ti.) **NOT** (news OR congres* OR abstract* OR book* OR chapter* OR dissertation abstract*).pt. **NOT** ((xenotransplant*).ti.) **AND** (english).lg **NOT** (liver* NOT (kidney* OR nephr*)).ti.

Appendix B. Data extraction of included articles (N=208)

**Author**	**Country**	**Study design**	**Sample** (N/ number of studies)	**Follow-up time points / months since donation Mean/Median (SD/IQR/range)**	**Postdonation psychosocial outcomes measured**	**Instruments**
Achille et al. 2007	Canada	Single-centre mixed-methods retrospective questionnaire survey	39 LKDs	Mean 44.14 (SD 25.2, range 5 - 89) months	Altruism, Gender stereotype, Family dynamics, Self-esteem, Decisional certainty, Satisfaction, Donation-specific questions	Self-reported Altruism Scale, Bem sex-role inventory, Family Environment Scale, Rosenburg self-esteem scale, Self-developed standardized questions
Al Breizat et al. 2020	Jordan	Multi-centre retrospective qualitative interview study	360 living kidney and liver donors	Range 14 days-7 years	Experiences before and after donation, motives, emotions	Not reported
Albertsmeyer et al. 2010	Germany	Single-centre retrospective questionnaire survey	69 LKDs	Mean 5.3 (SD 0.4) years	QoL	SF-36
Andersen et al. 2007	Norway	Multi-centre prospective RCT	122 LKDs	1, 6 and 12 months	QoL	SF-36, Quality of Life Scale-Norwegian
Baines et al. 2001	Scotland	Single-centre prospective qualitative interview study	7 LKD-recipient pairs	Immediately post-donation and 3, 6, 9, & 12 months post-donation	Changes in donor-recipient relationship	Not reported
Barnieh et al. 2021	Canada	Multi-centre prospective questionnaire survey	821 Directed and Nondirected LKDs	Approached pre-donation, and 3 & 12 months post-donation	Out-of-pocket costs and lost productivity costs, QoL, Anxiety, Depression	Standardized open and closed-ended self-developed questions , SF-36, BAI, BDI
Benzing et al. 2015	Germany	Single-centre retrospective questionnaire survey	55 Directed LKDs	Mean 6.2 (SD 2.8) years	QoL, Donation-impact	SF-36, Standardized self-developed questions

Bieniasz et al. 2018	Poland	Single-centre retrospective questionnaire survey	40 Directed LKDs	Mean 65.6 months	Psychological consequences of donation	SWLS, Situation Assessment Questionnaire, Health Behaviors Survey, Standardized self-developed questions
Binet et al. 1997	Switzerland	Single-centre retrospective questionnaire survey	23 LKDS, 23 recipients (11 LKDS completed questionnaires)	Mean 28 (SD 3) months (cohort characteristics)	Donor-recipient relationship, Coping with daily life, Fatigue	Standardized self-developed questions
Boyarsky et al 2014	USA	Single-centre mixed-methods retrospective questionnaire survey	1046 LKDs	Median 6.7 (range 3.7-11) years	Difficulty obtaining or changing health and insurance, experiences	Standardized open and closed-ended self-developed questions
Brown et al. 2008*	Canada	Single-centre retrospective qualitative interview study	12 LKDs	4-29 years	Experience of healthcare system	Interview guide
Brown et al. 2008*	Canada	Single-centre retrospective qualitative interview study	12 LKDs	4-29 years	Experiences, feelings and ideas on donation decision-making and psychosocial issues	Interview guide
Bruintjes et al. 2019	Netherlands	Single-centre retrospective questionnaire survey	512 LKDs	Mean 75.2 (SD 44.3) months	Pain, QoL	RAND SF-36, McGill Pain Questionnaire
Buell et al. 2008	USA	Multi-centre retrospective questionnaire survey	150 LKDs	Not reported	QoL	Functional Donor Survey, SF-36, general Health-Related QoL score
Burroughs et al. 2003	USA	Multi-centre prospective questionnaire survey	174 Directed LKDS, 174 recipients, 174 friends/relatives	Mean 6.67 (SD 4.6) years	Donation experience, decision-making, concerns, effect of donation on donor-recipient relationship	Standardized self-developed questions

Chen et al. 2004	Taiwan	Single-centre questionnaire survey	17 LKDs	Questionnaire: Not reported Clinical data: 6 months, 1 year, and 3 years	QoL	SF-36
Chen et al. 2015	China	Single-centre retrospective questionnaire survey	98 Directed LKDs	Mean 19.7 (SD 9.4) months	QoL, Depression, Anxiety	SDS, SAS, SSRS, MOS SF-36
Chen et al. 2016	Taiwan	Single-centre retrospective questionnaire survey	34 Directed LKDs	Mean 80.8 (SD 52.8) months	QoL, Depression, Anxiety, Concerns	SF-36, HADS, Donor Concerns scale
Clarke et al. 2013	UK	Multi-centre retrospective qualitative interview study	14 NDDs	Mean 15 (SD 9.12) months	Donor experiences	Interview guide
Clarke et al. 2006	N/A	Systematic review	35 studies	Range from 4 weeks to 9 years	Economic impact (direct and indirect costs)	
Clemens et al. 2006	N/A	Systematic review	51 studies; 5139 donors	Mean 4 (range 1 week to 37) years	Psychosocial health	
Clemens et al. 2011	Canada, Scotland, Australia	Multi-centre retrospective questionnaire survey	203 Kidney donors & 104 healthy nondonor controls	Donors: Mean 7 (SD 6) years & Non-donors: Mean 8 (SD 6) years	QoL, donation-related attitudes, psychiatric treatment	SF-36, 15D version 2.0, Feeling Thermometer (VAS), self-developed questions
Corley et al. 2000	USA	Single-centre retrospective questionnaire survey	55 LKDs	Range several months to 25 years	QoL, Attitude, Self-image	Donor Attitude Scale, Adult Image Scale, Ferrans and Powers Quality of Life Index, Ladder of Life, Marlowe-Crowne Social Desirability Scale

Dahm et al. 2006	Switzerland	Single-centre retrospective questionnaire survey	152 LKDs	Open group: Mean 69 (range 37-256) months & laparoscopic group: Mean 23 (range 12 – 51) months	Satisfaction with donation, attitudes and emotions	Standardized questions adapted from earlier studies
Davis et al. 2022	USA, Canada	Qualitative analysis of storytelling	82 LKDS, 36 recipients	Not reported	Challenges faced, coping strategies used, advice shared	Storytelling prompts
De Graaf Olson & Bogetti-Dumlao 2001	USA	Single-centre retrospective questionnaire survey.	62 LKDs	Mean 22.3 (range 2-48) months	Qol, General health, Follow-up	SF-36, Standardized self-developed questions
De Groot et al 2012	Netherlands	Single-centre retrospective questionnaire survey	N = 316	Mean 5.07 (SD 3.2, range 0.9 - 13.5) years	HRQoL, fatigue, societal participation	SF-36, MFI-20, Utrecht Scale for Evaluation of Rehabilitation-Participation
Dew et al. 2012	N/A	Conference workshop report	N/A	N/A	Psychosocial and socioeconomic issues	N/A
Dew et al. 2014	N/A	Review	Not reported	Range 0.9 - 48 years	Psychosocial outcomes, Financial impact	N/A
Dols et al. 2010	Netherlands	Multi-center prospective randomized controlled trial	94 living kidney donors	Median 6 (range 1-8) years	Long-term physical and psychosocial effects of donation after MIDN and LDN including HRQoL and fatigue	SF-36, SCL-90
Ehlers et al. 2017	N/A	Review	Not reported	N/A	Mental health, motivation among NDDs	N/A
Fallahzadeh et al. 2013	Iran	Single-centre retrospective questionnaire survey	144 LKDs (52 paid unrelated donors, 92 living related donors)	PUKDs: Mean 2.9 (SD 1.6) years & LRKDs: Mean 3.8 (SD 2) years	HRQoL	SF-36

Farahani et al 2016	Iran	Multi-centre retrospective qualitative interview study	16 related LKDs	Range <1 year to >5 years	Outcomes, feelings, attitude, relation with the recipient	Interview guide
Fathallah et al. 2021	Egypt	Single-centre retrospective questionnaire survey	50 Directed LKDs to pediatric recipients	Mean 4.9 (SD 2.78) years	QoL	WHOQOL-BREF
Fehrman-Ekholm et al. 2000	Sweden	Single-centre retrospective questionnaire survey	370 Donors and 1257 age and gender-matched comparison group	Mean 12.5 (SD 7.7) years	HRQoL, regret, impact on life, financial compensation	SF-36, self-developed statements
Feltrin et al. 2007	Italy	Multi-centre retrospective questionnaire survey	80 LKDs	Mean 16.2 (range 5-30) months	QoL, Donation experience	SF-36, Standardized self-developed questions
Fleishman et al. 2020*	USA	Multi-centre prospective questionnaire survey (KDOC study)	193 Donors and healthy controls	Pre-donation and 1, 6, 12, & 24 months post-donation	Pain management, pain expectancy, donation-specific pain, chronic postsurgical pain	LDEQ, The Pain Rating Index from the Short-Form McGill Pain Questionnaire, Pain Intensity Rating, Overall Pain Intensity
Frade et al. 2008	Portugal	Single-centre prospective questionnaire survey	32 LKDs	pre-donation and mean 18.8 (SD 12.8) months post-donation	QoL, Donor decision-making, motivation, donor-recipient relationship, outcome, pain, financial burden, quality of information, attitudes	Zung self-rating anxiety scale, SF-36, Kidney donor perceptions questionnaire
Frade et al. 2011	Portugal	Single-centre mixed methods retrospective questionnaire survey	45 Donors and 35 recipients	1 year	Donation perceptions	Self-developed questions

Frech et al. 2018	USA	UNOS/OPTN database analysis	9649 LKDs	At time of living donor registration and 6 months, 1 year, and 2 years post-donation	Return to paid employment, marital status	N/A
Friedersdorff et al. 2016	Germany	Multi-centre retrospective questionnaire survey	161 LKDs	ODN: Mean 94.3 (SD 39.3) months & LDN: 49 (SD 21) months	QoL	Standardized self-developed questions
Fry-Revere et al. 2020	Iran	Multi-center qualitative interview study	50 LKDs	Range, pre-donation to > 1 year post-donation	Pressure to donate, satisfaction, stigma	Interview guide
Fu et al. 2021	N/A	Systematic review	16 studies	Mean recall time 3 months - 10 (SD 10) years	Economic consequences of donation	N/A
Garcia et al. 2013	Brazil	Single-centre prospective questionnaire survey	50 LKDs	Mean 20 (SD 27) days pre-donation and 126 (SD 89) days & 445 (SD 164) days post-donation	QoL, Donor motivation, adequacy of information, pressure, recovery time, expenses, employment, overall experiences, sexual and emotional functioning, relationship with recipient, repeating donation	SF-36, Donor questionnaire developed in an earlier study
Garcia-Ochoa et al. 2021	Canada	Multi-centre prospective questionnaire survey	912 LKDs	Pre-donation and 3 & 12 months post-donation	QoL	SF-36, BDI, BAI
Garg et al. 2021	USA	NKR database analysis	326 LKDs who received reimbursement	N/A	Expenses reimbursement and amounts	N/A
Gibney et al. 2010	USA	UNOS/OPTN database analysis	10,021 LKDs	N/A	Health insurance status	N/A

Giessing et al. 2004*	Germany	Single-centre retrospective questionnaire survey	106 LKDs	Minimum 1 year follow-up. Median 75.3 (SD 66, range 12-226) months	QoL, Subjective complaints, source of information, influence on psychological and physical aspects, impact on social life, work and finances	SF-36, Giessen Subjective Compliants List, Standardized self-developed questions
Giessing et al. 2005*	Germany	Single-centre retrospective questionnaire survey	106 LKDs	Mean 75 (SD 66, range 12-226) months	QoL, Psychosomatic complaints, Experience of surgical techniques, importance of surgical technique, willingness to donate again	SF-36, Giessen Subjective Complaints List
Glotzer et al. 2013	USA	Single-centre retrospective questionnaire survey	83 LKDs, 116 incompatible donor candidates	Not reported	QoL, complaints (including chronic pain), donation related questions	SF-12, Standardized self-developed questions
Greif-Higer et al. 2008	Germany	Single-centre prospective study	17 donors and their recipients	1 - 6 years	Donor-recipient relationship	Clinical interview (ICD 10), HADS-D, Mainz interview for LOD
Gross et al. 2013*	USA	Multi-centre retrospective questionnaire survey (RELIVE study)	2455 Donors and US population databases (n = 3828, n = 988)	Mean 17 (SD 10, range 5 - 48) years	HRQoL, self-rated health status, impact on general health	SF-36, self-developed questions
Haljamae et al. 2003	Sweden	Single-centre retrospective qualitative interview study	10 LKDs who's recipient had lost the graft	>3 years after early recipient graft loss or death of recipient (range 1 week – 4 years)	Experiences when the graft is lost	Not reported
Hamama-Raz et al. 2020	Israel	Single-centre retrospective questionnaire survey	78 NNDs, 396 nondonors	Mean 2.3 (SD 1.3, range 0.5 – 6) years	Subjective health status, Death anxiety, Psychological distress	Standardized self-developed item, Death Anxiety Questionnaire, Kessler's K6
Han et al. 2010	Korea	Single-centre retrospective	205 LKDs	Not reported (>1 month)	QoL, Pain, Cosmetic aspects	SF-36, Standardized self-developed questions

		questionnaire survey				
Han et al. 2017	Singapore	Single-centre prospective questionnaire survey	82 LKDs	Median 5.7 years	QoL	SF-36
Heck et al. 2004	Germany	Single-centre retrospective qualitative interview study	31 Donor-recipient couples	Median 2.5 (range 0.7 - 5.9) years	Psychosocial effects of transplantation and donation	Interview manual
Heidary et al. 2009	Iran	Multi-centre retrospective questionnaire survey	600 unrelated compensated LKDs	Not reported	Feelings after donation, attitude towards keeping in touch with recipient	Standardized self-developed questions
Hildebrand et al. 2014	USA	Single-centre retrospective questionnaire and focus group interview study	76 LKDs (survey), 21 LKDs (focus groups)	1 – 6 years	Psychological reaction after donation, decision-making, positive and negative postdonation experiences	Standardized self-developed questions, interview script
Hirvas et al. 1976	Finland	Single-centre interview study	64 LKDs, 10 nephrectomy patient controls	Pre-donation, and 6 months – 6 years post-donation	Emotional problems attributed to the procedure	Not reported
Hirvas et al. 1980	Finland	Single-centre interview study	16 LKDs	Pre-donation and 1 year post-donation	Prediction of psychic traumatization	Not reported
Ho et al. 2022	USA	Qualitative analysis of storytelling	82 LKDs, 36 recipients	Not reported	Experiences, emotional changes	Prompts on storytelling platform
Hoda et al. 2010	Germany	Single-centre retrospective questionnaire survey	44 LKDs	Median 21.1 (SD 6.2, range 5 - 40) months	QoL, scar pain, stressfulness of donation, life changes associated with donation, self-esteem	SF-36, standardized self-developed questions
Holscher et al. 2018*	USA	Single-centre retrospective questionnaire	825 Donors	Median 6 (IQR 10) years	Depression, anxiety, regret, psychotropic medications, difficulty changing or	GAD-2, PhQ-2, self-developed questions, ZIP code

		survey (WHOLE-donor study)			obtaining new health insurance or life insurance, socioeconomic status	
Isotani et al. 2002	Japan	Multi-centre retrospective questionnaire survey	69 Directed donors	Mean 6.95 (SD 3.4, range 0.3 - 14) years	QoL, donation-related stress, satisfaction, expenses incurred, physical changes, and pre-existing factors such as relationship to the recipients	SF-36, self-developed questions
Jackobs et al. 2005	Germany	Single-centre retrospective questionnaire survey	98 LDN LKDS, 41 MIDN LKDs	Range 3 – 69 months; LDN: Mean 49 (SD 16) months & MIDN: Mean 13 (SD 6) months	QoL, donors' attitude towards kidney donation, financial burden, pain, cosmetic satisfaction, duration of sick leave	SF-36, Standardized self-developed questions
Jacobs et al. 2015*	USA	Multi-centre retrospective questionnaire survey (RELIVE study)	2455 Donors	Mean 17 (SD 10, range 5 - 48) years	Donor health status, recovery time, social support, motivation, overall experience, donor-recipient relationship, financial experience	Standardized questions adapted from earlier studies
Jacobs et al. 2019	USA	Single-center retrospective questionnaire survey	77 Nondirected donors	Mean 6.7 (SD 4) years	Motivation, Support from others, Associated stress, Regret, Impact on life, Financial resources, Communication and anonymity, Preferences for NDD policies	Standardized questions adapted from earlier studies
Janki et al. 2015	Netherlands	Single-centre prospective questionnaire survey	97 Kidney donors	10 years	QoL, fatigue	SF-36, MFI-20
Jaseanchiun et al. 2012	Thailand	Single-centre retrospective questionnaire survey	49 LKDs	Not reported	QoL	SF-36

Jawad et al. 2003	Pakistan	Single-centre retrospective analysis of medical records	450 LKDs	Not reported	Social and psychological impact after donation	Not reported
Johnson et al. 1999	USA	Single-centre retrospective questionnaire survey	524 Kidney donors	Range <1 year - >5 years	QoL, donation information, donation stress, expenses incurred, physical changes, emotional changes	SF-36, standardized self-developed questions
Jorden et al. 2004	Germany	Single-centre retrospective study	112 LKDs	Mean 11.2 (SD 7.5) years	Psychological variables (symptoms, health behavior, health consciousness)	Not reported
Joshi et al. 2013	India	Multi-centre prospective mixed methods study	30 LKDs, 30 recipients	pre-donation and <5 years post-donation	QoL, physical health, occupation, marital and family relations, sociorecreational, spiritual, emotional	KDQOL SF1.3, interview schedule
Jowsey & Schneekloth 2008	N/A	Review	Not reported	N/A	Psychosocial evaluation, outcomes, nondirected donors, paired-exchange, living liver & liver donors	N/A
Jowsey et al. 2014*	USA	Quantitative record review & retrospective questionnaire survey (RELIVE study)	2455 Kidney and lung donors. Matched control group from National Health and Nutrition Examination Survey (NHANES)	Not reported	HRQoL, predonation psychiatric disorders and treatment, postdonation depression, psychotropic medication use, optimism, donation experience, attitudes about donation and donation-related relationships	PHQ-9, LOT-R, SF-36, standardized self-developed questions
Kadioglu et al. 2012	Turkey	Single-centre retrospective questionnaire survey	30 LKDs, 30 recipients	Range 3 months - 1 year	Depression, Anxiety, Dyadic adjustment, psychological help	BDI, HADS, Dyadic Adjustment Scale, standardized self-developed questions

Karagöl 2019	Turkey	Single-centre retrospective questionnaire survey	36 LKDs, 15 living liver donors	Not reported	QoL, depression, anxiety	SF-36, BDI, BAI
Karagöl & Kaya 2022	Turkey	Single-centre retrospective questionnaire survey	71 LKDs, 22 living liver donors	Not reported	Early Maladaptive Schemas, depression	Young Schema Questionnaire-Short Form 3, BDO
Katvan et al. 2022	Israel	Multi-centre retrospective questionnaire survey	131 directed LKDs, 115 nondirected LKDs	Mean 470.16 (SD 287.11, range 21 - 1095) days	Post-donation recalled pain memory	Standardized self-developed questions
Kaul et al. 2022	India	Single-centre retrospective questionnaire survey	506 LKDs	Mean 27.6 (range >6 months – 60) months	QoL, Depression, Anxiety, donor experience, recovery, current psychological status, motivation, interpersonal relationship with the recipient, support, financial effects, and long-term outcomes	SF-26, BDI, HADS, Standardized self-developed questions
Keys et al. 2019	USA	Single-centre database analysis	21 LKDs	Mean 50.7 (SD 1.9) years	QoL ≥50 years postdonation	SF-36
Klarenbach et al. 2014	Canada	Multi-centre prospective questionnaire survey	100 Directed donors	3 and 12 months	Economic consequences	Direct out-of-pocket costs, home productivity cost, workforce productivity cost
Klop et al. 2013	Netherlands	Single-centre prospective questionnaire survey	135 LKDs > 60 years, 366 LKDs < 60 years	1, 3, 6, and 12 months	QoL in elderly versus younger donors	SF-36
Klop et al. 2018	Netherlands	Single-centre prospective questionnaire survey	501 Directed and nondirected donors (NDDs)	Pre-donation and 1, 3, 6, and 12 months post-donation	QoL, Pain severity	SF-36, VAS

Kobayashi et al 2019	Japan	Single-centre retrospective questionnaire survey	195 Directed donors	Low satisfaction group: Mean 57.3 (range 3.8–205.3) months & Non-low satisfaction group: Mean 58.2 (range 1.0–833.3) months	Satisfaction, QoL, depression, optimism, recipients' clinical characteristics, perceptions of results and procedure,	CSQ-8, SF-36, SDS, LOT, standardized questions adapted from earlier study
Kok et al. 2006	Netherlands	Single-centre prospective questionnaire survey	45 MIDN LKDS, 55 LDN LKDs	Pre-donation and 1, 3, 6, and 12 months post-donation	QoL, fatigue	SF-36. MFI-20
Kowal et al. 2021	Poland	Multi-centre retrospective mixed-methods study	25 LKDs	Mean 40.12 (SD 8.378, range 22-50) months	Body image and esteem	Body Esteem Scale, interview guide
Krishnan et al. 2020	UK	Multi-centre quantitative database analysis	9750 Donors (UK Transplant Registry) and 19071 nondonor comparison (The Health Improvement Network (THIN) database)	1, 2, 5, 10, and up to 15 years	Mortality and morbidity, including depression	Depression instrument not reported
Kroencke et al. 2012	Germany	Single-centre prospective questionnaire survey	79 LKDs	Pre-donation, and 3 months and 1 year post-donation	QoL, depression, anxiety	SF-36, HADS
Ku 2005	N/A	Review	11 studies including 1683 LKDs	Range of multiple studies: Mean 4 months - mean 12.5 (SD 7.7) years	QoL	SF-36

Langenbach et al. 2009	Germany	Single-centre retrospective qualitative interviews	11 Directed donors	Not reported	QoL, subjective evaluation of donation	Not reported
Lentine et al. 2012	USA	Quantitative Database analysis	4650 Directed donors. Matched nondonor insurance beneficiaries.	Median duration from donation to start of insurance benefits: 4.9 (range 1.7 - 8.7) years	Depression, donor characteristics, recipient clinical outcomes	Depression diagnosis registered with health insurance
Lentine et al. 2019	N/A	Review	Not reported	N/A	Psychosocial outcomes	Not reported
Li et al. 2017	USA	Single-centre retrospective questionnaire survey	110 LKDs, 31 living liver donors	Mean 5 (SD 3.9, range 0 - 13) years	QoL, pain, donation experiences, suggested improvements	Standardized self-developed questions
Lim et al. 2022	N/A	Qualitative systematic review	12 studies, 129 unspecified kidney donors and 26 living liver donors	Not reported	Donor perspectives or experiences	Semi-structured interview and focus group guides
Lima et al. 2006	Brazil	Single-centre retrospective questionnaire survey	100 LKDs, 100 community controls	Mean 8 (SD 5) years	QoL	SF-36
Liu et al. 2018	Taiwan	Single-centre retrospective questionnaire survey	41 directed LKDs	Mean 59.12 (SD 49.90) months	QoL, positive and negative affect, decision-making, regret	PANAS, SF-12, , Effectiveness Decision SubscaleDecision Regret Scale
Liu et al. 2021	China	Meta-analysis review	9 studies including 802 Chinese LKDs	Not reported	QoL	SF-36
Lopes et al. 2011*	Portugal	Single-centre prospective questionnaire survey	45 directed LKDs, 35 recipients	pre-donation and ≥1 year post-donation	Depression, Anxiety	Zung Self-rating Anxiety Scale, Zung Self-rating Depression Scale

Lopes et al. 2013*	Portugal	Single-centre prospective questionnaire survey	45 directed LKDs, 35 recipients	Pre-donation and ≥1 year post-donation	QoL	SF-36
Lumsdaine et al. 2005	UK	Multi-centre prospective questionnaire survey	40 LKDs, 35 recipients	Pre-donation, and 6 weeks and 1 year post-donation	QoL, family dynamics	WHOQOL-BREF, Standardized self-developed questions
Lunsford et al. 2007	USA	Single-centre retrospective interview study	18 LKDs	Not reported	Racial differences in donation experiences	Interview script
Maghen et al 2018*	USA	Multi-centre retrospective interview study	30 NNDs	Not reported	Influence of spirituality and religiosity on donation experience	Interview guide
Maghen et al. 2021*	USA	Multi-centre retrospective interview study	31 NNDs	Not reported	Financial concerns	Interview guide
Maglakelidze et al. 2011	Georgia	Single-centre retrospective questionnaire survey	57 directed LKDs, 120 matched controls, 52 renal tumor nephrectomy patients	Mean 32 (range 4-57) months	QoL, Mood, Subjective complaints	SF-36, Zerssen's mood Scale, Giessen Subjective Complaints List
Manera et al. 2017	Australia, Canada	Qualitative multicentre retrospective focus group interview study	123 Directed and nondirected donors	Range 6 weeks - 16 years	Experiences and expectations of follow-up and aftercare	Focus group question guide
Maple et al. 2014	UK	Multi-centre retrospective questionnaire survey and database analysis	80 Directed and 110 nondirected donors (NDDs)	UKD: Mean 1.3 years & SKD: Mean 2.6 years	Well-being, Stress, Distress, Depression, Anxiety, Physical health-related QoL Self-esteem, Optimism Social Support, Social Comparison, Markers of altruism, Medical history	Office of National Statistics Wellbeing questions, SWLS Perceived stress scale, GHQ-12, PHQ-2, STAI-6, SF-12

					Postoperative recovery<br>Clinical outcomes	Rosenburg self-esteem scale<br>LOT-R, Multidimensional scale of perceived social support,<br>Social comparison scale,<br>10-item Personality inventory,<br>Standardized self-developed questions
Maple et al. 2017	UK	Single-centre quantitative prospective study	93 Directed and nondirected donors (NDDS)	pre-donation, and 3 and 12 months post-donation	Well-being, Distress, Mood Stress, Physical health-related QoL, Life satisfaction,<br>Self-esteem, Anxiety Optimism, Social support, Social comparison	Office of National Statistics Wellbeing questions, GHQ-12<br>PHQ-2, Perceived stress scale,<br>SF-12, SWLS, Rosenburg self-esteem scale STAI-6, LOT-R, Multidimensional scale of perceived social support, Social comparison scale<br>Standardized self-developed questions
Maple et al. 2020	N/A	Review	Not reported	N/A	Benefits and controversies of unspecified donation	N/A
Massey et al. 2010	Netherlands	Single-centre retrospective mixed-methods study	24 NDDs	Mean 2.3 years (range 3 – 97 months, median 18 months)	DSM-IV classification, Psychological symptoms, Impact of donation, Experiences, Satisfaction	MINI-plus, SCL-90, VAS<br>Self-developed questions
Massey et al. 2022*	Netherlands	Single-centre retrospective mixed-methods study (MEGA study)	114 NDDs	Median 76.5 (range 23 – 178) months	Positive mental health, Psychological symptoms, Psychiatric diagnoses	D-MHC-SF, SCL-90, MINI-plus

Menjivar et al. 2018	Spain	Single-centre retrospective questionnaire survey	332 donors	Mean 4 (SD 2.2) years	Donor satisfaction, Perception of recipient health status, Repentance of having donated Feeling of recipient dependency, Feeling of being more valued Current relationship with recipient, Feeling that discharge was premature, Economic losses, Willingness to donate again, Recommendation of living donation	ESS, VAS
Menjivar et al. 2020	Spain, Germany, Sweden, France, Portugal	Multi-centre prospective questionnaire survey	75 Directed donors	Mean 12.78 (SD 3.44) months	QoL, Mental health status Coping strategies, Personality, Socio-economic status	SF-36, ACSA, HADS, LOT-R, SOCS, EQP-RA, ELSA
Messersmith et al. 2014*	USA	Multi-centre questionnaire survey (RELIVE study)	2455 Donors	Mean 17.3 (SD 9.8, range 5 - 48) years	Satisfaction with life, physical health, optimism, social support, evaluation of overall donation experience	SWLS, LOT-R, SF-36, Structured self-developed questions
Meyer et al. 2016*	Norway	Single-centre retrospective questionnaire survey	217 Directed donors	Median 10 (range 8.5 – 12) years	QoL, Fatigue, regret, perceived recipient outcome, economic problems, follow-up, recognition from others, use of analgesia, satisfaction with life before and after donation	SF-36, MFI-20, Structured self-developed questions

Meyer et al. 2017*	Norway	Single-center retrospective questionnaire survey	202 LKDs	Mean 11.3 (SD 1.5, range 6 – 15, median 11) years	QoL 10 years postdonation, Fatigue, Donation-specific factors	SF-36, MFI-20, Structured self-developed questions
Meyer et al. 2017	Norway	Single-centre retrospective interview study	16 directed LKDs	Range 10 – 14 years	Subjective meaning and experiences 10 years post donation	Interview guide
Minnee et al. 2008	Netherlands	Single-centre prospective questionnaire survey	105 LKDs	SF-36: Pre-donation and 1, 2, 4 weeks, 3 months, 6 months, and 12 months post-donation; MFI-20: pre-donation and 1, 3, 6, and 12 months post-donation; VAS: pre-donation and 1, 2, 3, 7, 14, and 28 days post-donation	QoL, fatigue, pain	SF-36, MFI-20, VAS
Mjoen et al. 2011	Norway	Single-centre retrospective questionnaire survey	1414 LKDs	Median 12.7 (range 1.1 - 42.9) years	QoL, Reconsideration of donation	SF-36, Structured question from earlier studies
Morgan & Ibrahim 2011	N/A	Review	Not reported	N/A	QoL	SF-12/36
Neuhaus et al. 2005	Switzerland	Single-centre retrospective questionnaire survey	20 parental LKDs and partners	Median 3 (range 1.5 - 8.3) years	Psychosocial impact of donation	Structured self-developed questions
Nohre et al. 2018	Germany	Single-centre retrospective	361 LKDs	Range 1 - 38 years	Partnership status and satisfaction	Quality of Marriage Index

		questionnaire survey				
Oguten et al. 2019	Turkey	Single-centre retrospective questionnaire survey	208 directed LKDs	Mean 4.55 (SD 2.5, range 1-11) years	Subjective evaluations, depression symptoms, anxiety symptoms, contentment with life	BDI, BAI, Contentment with Life Assessment Scale (CLAS), Structured self-developed questions
O'Keeffe et al. 2018	N/A	Systematic review & meta-analysis	52 studies, 118,426 living donors and 117656 nondonors	1-24 years	HRQoL	N/A
Oliveira et al. 2011	Portugal	Single-centre retrospective interview study	30 LKDs	6 – 30 months	Satisfaction, pain, positive and negative aspects	Questionnaire guide
Ong et al. 2021	N/A	Systematic review	62 studies on living kidney, liver, bone marrow, uterus and lung donors	3 months – 48 years	Depression, anxiety	N/A
Owen et al. 2010	Scotland	Single-centre retrospective questionnaire survey	81 LKDs	Up to 10 years	Chronic pain, QoL	Short Form Brief Pain Inventory (sf-BPI), EuroQoL (EQ5D), Selfreport Leeds, Assessment of Neuropathic Symptoms and Signs (s-LANSS)
Özçurumez et al. 2004	Turkey	Single-centre retrospective questionnaire survey	18 LKDS, 49 recipients	Donors: Mean 35.6 (SD 29.3, range 1 – 120) months Recipients: Mean 37.1 (SD 31.2, range 2 - 136) months	Psychiatric history, tobacco and alcohol consumption, drug abuse, relationship with recipients, satisfaction with QoL	Structured self-developed questions
Padrão et al. 2009	Brazil	Single-centre retrospective	69 LKDs, 68 controls	>1-25 years	QoL	SF-36, WHOQOL-BREF

		interview and questionnaire survey				
Park et al. 2022	Korea	Multi-centre retrospective matched cohort study	1285 LKDs, 1285 matched controls	1, 2, 3, 4, & 5 years	Economic status	Health insurance fees, change in employment status
Pawłowski et al. 2020	N/A	Review	15 articles, donors and recipients	1 - 484 months	QoL, symptoms of depression and anxiety	N/A
Pollmann et al. 2017	Germany	Single-centre retrospective questionnaire survey	315 LKDs	Mean 7.1 years (SD 5.2)	Personality, depression, anxiety, fatigue, donation-specific questions (relationship, regret, willingness to donate again)	NEO-FFI, PHQ-9, GAD-7, MFI-20, Structured self-developed questions
Pronk et al. 2023*	Netherlands	Single-centre retrospective qualitative interview study (MEGA study)	106 NDDs	Median 71.50 months (range 23–153)	Motivation, Impact of donation on mental health	Interview guide
Pronk et al. 2023*	Netherlands	Single-centre retrospective qualitative interview study (MEGA study)	106 NDDs	Median 71.50 months (range 23–153)	Donor experiences	Interview guide
Ralph et al. 2017	N/A	Qualitative review	40 studies including 889 LKDs & 551 recipients	1 week – 25 years	Views on donor-recipient relationship	N/A
Ralph et al. 2019	Australia	Multi-centre prospective qualitative interview study	16 LKDs, 16 recipients	Pre-donation & 11-14 months post-donation	Expectations and perspectives of the donor-recipient relationship	Interview guide
Rana et al. 2010	UK	Single-centre retrospective	56 LKDs	12 weeks – 6 years	Donation experience (pain, information provision, fitness & work, costs,	VAS, Structured self-developed questions

		questionnaire survey			recommendation of donation)	
Reese et al.2015	N/A	Review	Not reported	N/A	QoL, Financial consequences	Not reported
Roberts & West-Wooding 2017	Trinidad & Tobago	Single-centre retrospective questionnaire survey	43 LKDs	3 months – 5 years	Pain, overall experience, willingness to donate again, recommendation of donation	Unstandardized questionnaire
Rodrigue et al 2006	USA	Single-centre retrospective interview study	40 ODN & 44 LDN LKDs	Mean 70.5 (SD 50.1) months	Return to functional status, financial impact, current health-related quality of life (SF-36 Health Survey11), and overall satisfaction with the donation experience	SF-36
Rodrigue et al. 2011	USA	Multi-centre retrospective survey study	39 NDDs and 52 traditional /directed donors	Median 5 (range 1-12) years	Motives, altruistic behaviours, Psychosocial outcomes, functional outcomes, QoL, Experiences and decision stability	LDEQ, SF-36, Standardized self-developed questions
Rodrigue et al. 2014	USA	Multi-centre prospective questionnaire survey	133 LKDs	1, 6, 12, and 24 months post-donation	Perceived benefit	LDEQ, SF-36 (recipients)
Rodrigue, Vishnevsky et al. 2015	USA	Single-centre prospective questionnaire survey	123 LKDs	12 & 24 months	Patient-Reported Outcomes: physical and psychosocial consequences of donation, donation satisfaction, and donation decision regret	SF-36, Standardized self-developed questions
Rodrigue & Fleishman 2016	USA	National UNOS/OPTN database analysis	53,724 living kidney donors	6 weeks, 6, 12, & 24 months	Health insurance	Health insurance status at time of donation, health risk score (obesity, smoking, hypertension, eGFR <60, proteinuria)

Rodrigue, Schold et al 2016*	USA	Multi-centre prospective questionnaire survey (KDOC study)	182 living kidney donors	1, 6, & 12 months	Financial burden	Direct costs (e.g. transportation), indirect costs (e.g. hours work missed), financial assistance
Rodrigue et al. 2018*	USA	Multi-centre prospective questionnaire survey (KDOC study)	193 living kidney donors and 20 health controls	1, 6, 12, and 24 months	Mood, body image, fear of kidney failure, life satisfaction, decisional stability	Profile of Mood States, Fear of Kidney Failure questionnaire, Body Image Scale, SWLS, Standardized self-developed questions
Rodrigue et al. 2020*	USA	Multi-centre prospective questionnaire survey (KDOC study)	193 living kidney donors and 20 health controls	Pre-donation and 1, 6, 12, and 24 months post-donation	Fatigue, predonation BMI, BP, smoking, substance abuse, mood disturbance, fear of kidney failure, physical activity	FSI, SF-36 Vitality scale, Profile of Mood States, Fear of Kidney Failure questionnaire, self-report questions
Sahay et al. 2007	India	Single-centre retrospective questionnaire survey	50 LKDs	Mean (Range) = 63 (3-264) months	QoL, regret	Not reported
Schnitzbauer et al. 2007	Germany	Single-centre prospective questionnaire survey	36 ODN & 34 MIDN LKDs	Pre-donation, 1 week, 3 and 12 months	QoL	SF-36
Schover et al. 1997	USA	Multi-centre retrospective questionnaire survey	167 living kidney donors	1-12 years	Quality of Life, perceived medical status of kidney and recipient, ease of deciding to donate, emotional pressure to donate, emotional closeness to recipient, conflict with recipient or family before and after donation, perceived medical and emotional recovery, worries about	Medical Outcomes Study Short-Form Health Survey (MOS-20), questions developed in previous studies

					impact on health, impact on finances, perceived gratitude, perceived effort by recipient to stay healthy, donation decision making	
Serur et al. 2014	USA	Single-centre retrospective questionnaire survey	34 traditional LKDs, 30 'chain' LKDs	1-6 years	Psychosocial and functional outcomes, coercion	LDEQ, MacArthur Admission Experience Survey-short form
Serur et al. 2015	USA	Single-centre retrospective questionnaire survey	9 community-solicited NDDs, 16 traditional NDDs	Mean 24 months (range 3-72)	QoL, coercion	SF-36, MacArthur Admission Experience Survey-short form
Serur et al. 2015	USA	Single-centre retrospective questionnaire survey	11 spouse donors, 31 non-spouse donors	Not reported	Effect of donation on marriage	Revised Dyadic Adjustment Scale
Shakya et al. 2016	Nepal	Single-centre retrospective questionnaire survey	59 LKDs, 59 matched reference group	Median 3.67 years (IQR 1.75–5.67)	QoL, donation-related questions	SF-36, Standardized self-developed questions
Sharma & Enoch 1987	UK	Single-centre retrospective clinical interview study	13 LKDs, 11 nondonors	5-10 years	Psychiatric disorders	Present State Examination
Sharp et al. 2010	UK	Single-centre retrospective questionnaire survey	17 LKDs	Mean 1.86 months (SD 0.97)	QoL, decision-making, donation experiences	SF-36, Donor Decision Control Scale, Living Donor Survey
Shaw & Bell 2015	New Zealand	Multi-center retrospective qualitative interview study	19 directed LKDs, 6 NDDs	Not reported	Experiences of financial incentives and reimbursement for costs	Standardized self-developed questions
Shi et al. 2023	China	Single-centre retrospective	122 LKDs	Median (IQR) = 29.5 (10.75 - 53) months	QoL, Depression, Anxiety	WHOQOL-BREF, PHQ-2, GAD-2

		questionnaire survey				
Shrestha et al. 2008	UK	Single-centre retrospective questionnaire survey	66 LKDs, 38 controls	Mean 4.6 years (range 3 months - 27 years)	QoL	SF-36
Skaczkowski et al. 2023	Australia	Retrospective qualitative interview study	17 LKDs	Mean (SD) = 6 (6) years. Range = 0 – 24 years	Experiences of rural donors, support services	Topic guide
Slakey et al. 2008	USA	Single-centre retrospective telephone survey	81 LKDs	> 12 months follow-up. Mean 60.3 months (SD 17.3)	Experiences and psychosocial issues postdonation	Standardized self-developed questions
Slinin et al. 2016	N/A	Systematic review	45 articles	> 5 years	Psychosocial outcomes	N/A
Smith et al. 2004	Australia	Single-centre prospective psychosocial (questionnaire) assessment	48 living kidney donors	Pre-donation & 12 months post-donation	Psychosocial functioning, QoL	PHQ-58, SF-36
Sommerer et al. 2015*	Germany	Single-centre retrospective questionnaire survey	295 living kidney donors	Median 77 (range 24–484) months	Psychosocial and physical outcomes	SF-36, MFI-20, PHQ-9 for depression, PHQ-15 for somatisation,
Sommerer et al. 2018*	Germany	Single-centre retrospective questionnaire survey	211 living kidney donors	Mean 9.7 years (SD 5.2)	HRQoL, gender	SF-36, MFI-20, PHQ-9 for depression
Soneji et al. 2008	N/A	Review	Not reported	Not reported	Long-term psychological effects of donation and QoL	N/A
Suwelack et al. 2022	Germany	Multi-centre prospective questionnaire survey (SoLKiD study)	336 LKDs	Pre-donation and 2, 6 and 12-months post-donation	QoL, Fatigue, Mental disorders	SF-36, MFI-20, PHQ-9 for depression, PHQ-15 for somatisation

Tanriverdi et al. 2004	Turkey	Single-centre retrospective questionnaire survey	18 LKDs, 49 recipients, 45 healthy controls	Mean 35.6 months (SD 29.3, range 1 - 120 months)	QoL , depression, anxiety	SF-36, BDI, BAI
Taskintuna et al. 2009	Turkey	Single-centre retrospective questionnaire survey	35 LKDs, 69 recipients, 45 healthy controls	Mean 33.21 (SD 29.56, range < 1 - 120) months	QoL , depression, anxiety	SF-36, BDI, BAI
Tellioglu et al. 2008	Turkey	Single-centre retrospective questionnaire survey	55 LKDs	Mean 55.1 months (SD 47.2, range 12–168)	QoL	SF-36
Thomas et al. 2021	N/A	Review	NDD	Not reported	Psychosocial consequences, psychological risks	N/A
Thys et al. 2015	N/A	Systematic review	23 studies on parental donors, pediatric recipients	< 1 year to > 10 years	Psychosocial impact on donors, recipients, and family	N/A
Timmerman et al. 2013	Netherlands	Single-centre prospective questionnaire survey	NDD	Pre-donation median 9 months (range 2-13). Post-donation median 19 months (range 3-36)	Psychological functioning	SCL-90
Timmerman et al. 2015*	Netherlands	Single-centre prospective questionnaire survey	135 living kidney donors and matched general population controls	Pre-donation baseline, 6, 9 and 15 months	Mental health and well-being	BSI, D-MHC-SF,
Timmerman, Laging et al. 2016*	Netherlands	Single-centre prospective questionnaire survey	145 living kidney donors	2.5 months pre-donation, 3 and 12 months post-donation	Donors' medical complications Recipients' medical complications, Mental health	BSI, D-MHC-SF, PANAS

Timmerman, Timman et al. 2016*	Netherlands	Single-centre prospective questionnaire survey	151 living kidney donors	2.5 months pre-donation, 3 and 12 months post-donation	Appraisals, expectations, knowledge, social support, coping, life events, mental health, relationship with recipient	BSI, D-MHC-SF, DASS – stress subscale, COPE-easy, SSL-I, SSL-D, self-developed items on appraisals and life events
Tomer et al. 2021	USA	Database analysis	2108 LKDs, 2108 matched nephrectomy controls	5 year incidence & 5 year relative risk	Depression diagnosis	ICD-9 codes
Tong, Chapman et al. 2012	N/A	Systematic review	26 studies on 478 living donors	1 week – 29 years	Motivation to donate and experiences of donation	Thematic synthesis
Tong, Craig et al. 2012	New Zealand	Single-centre retrospective qualitative interview study	18 non-directed living kidney donors	Mean 42 (range 5-147) months	Motivations and experiences of non-directed donors	Interview guide
Tumin et al. 2014	Malaysia	Single-centre retrospective questionnaire survey	80 LKDs, 80 healthy matched controls	Not reported	QoL	SF-36
Tushla et al. 2015	N/A	Consensus conference report	N/A	N/A	Financial burden (direct and indirect costs)	N/A
Ummel & Achille 2015	Canada	Single-centre retrospective qualitative interview study	5 LKDs, 5 recipients	Mean 21 (SD 9, range 8-32) months	Donation/transplantation experience as a dyad	Interview schedule
Van Pilsum et al. 2020*	USA	Single-centre retrospective qualitative study (WHOLE study)	56 living kidney donors	Median 9.4 (IQR 6.4–12.4) years	Benefits experienced	Interview guide
Vernadakis et al. 2021	Greece	Single-centre retrospective cohort & questionnaire survey	117 LDN & 135 ODN LKDs	LDN: Median 11 (SD 7, range 1-26) months & ODN: median 42 (SD 13,	QoL	SF-36

				range 5-71) months		
Vlaovic et al. 1999	Canada	Single-centre retrospective questionnaire survey	104 LKDs	Not reported	Psychological well-being, impact of donation	Standardized self-developed questions
Von Zur-Muhlen et al. 2017		Single-centre retrospective questionnaire survey	387 LKDs	Mean 11 (SD 7, range 1–33) years	Relationship to recipient, experiences of donation, long-term effects and outcomes, gender differences	Standardized self-developed questions
Wadström et al. 2019	Sweden	Multi-centre retrospective questionnaire and interview study	24 NDD	6 years	Experiences, views on anonymity, psychosocial and medical status at follow-up (depression, anxiety)	HADS, self-developed standardized questions
Wahba et al. 2021	Germany	Single-centre prospective questionnaire survey	96 LKDs (28 AA & 68 HARP)	12 months post-donation and yearly thereafter	QoL, Fatigue, Aniety, Depression	SF-36, MFI-20, HADS
Walsh 2004	Northern Ireland	Single-centre retrospective interview study	8 LKDs	Not reported	Psychological experiences and meaning of decision-making processes	Interview guide
Watson et al. 2013	USA	Multi-centre prospective questionnaire survey	1030 LKDs	6 months post-donation, yearly thereafter	QoL	SF-36
Weitz et al. 2006	N/A	Review	Not reported	Not reported	Psychological impact, QoL, regret	Not reported
Westlie et al. 1993	Norway	Multi-centre retrospective questionnaire survey	494 LKDs, 74977 healthy controls	6.7 years (1-19 years)	QoL	Standardized questions from an earlier study
Wiedebusch et al. 2009	Germany	Single-centre retrospective	131 LKDs	Mean 61.3 months (SD 44.7), range 2-280	QoL, Depression, Anxiety, Coping	SF-36, HADS, Freiburg Questionnaire of Coping with Illness

		questionnaire survey				
Williams et al. 2009	Australia	Single-centre retrospective qualitative study	18 living kidney donors	12 months to 11 years	Experiences of donation	Interview guide
Wirken et al. 2015	N/A	Systematic review & Meta-analysis of prospective studies	34 studies on 3201 living kidney donors	1 month – 6 years	HRQoL	BDI, EQ-5D, HADS, MFI-20,<br>SAS, SCL-90, SDS, SF-36, SSQ, STAI, VAS, WHOQOL-BREF
Wirken et al 2019	Netherlands	Multi-centre prospective study	230 LKDs	Pre-donation, 6 months and 12 months post-donation	HRQoL, donor perceived and recipient-related consequences of donation, regret	RAND SF-36, CIS, VAS, self-developed standardized questionnaire on donor-perceptions, Decision Regret Scale
Wirken et al. 2022	Netherlands	Multi-centre prospective study	588 potential donors, 361 who donated, 230 with prospective data	Pre-donation, 6 months and 12 months post-donation	Risk assessment by transplant professionals, HRQoL, Personality, Fatigue, Social support, Donor cognitions, Social-relational functioning, recipient functioning	SF-36, NEO, Checklist Individual Strength-Fatigue scale, Inventory for Social Resilinece-Perceived social support scale, Donation Cognition Instrument, LDEQ, Interpersonal Sensitivity Measures, self-developed standardized questions
Woldemichael et al. 2021	Ethiopia	Single-centre retrospective questionnaire survey	64 directed LKDs	Not reported	Psychological well-being	Ryff Psychological well-being scale
Wolters et al. 2003	Germany	Single-centre retrospective questionnaire survey	58 LKDs	Mean 28 (SD 18) months	Financial risk	Not reported

Yang et al. 2007	N/A	Systematic review	23 studies including 2067 LKDs	Mean 6 months to 7.4 years post-donation (range 3 months - 31 years)	Insurability	N/A
Yasumura et al. 1988	Japan	Single-centre retrospective questionnaire study	157 LKDs	18 months to 16 years and 2 months	Pain	Not reported
Yucetin et al. 2015	Turkey	Single-centre retrospective questionnaire survey	184 LKDs	Mean 20.84 months (SD 15.50, range 6-67)	Post-traumatic growth	Posttraumatic Growth Inventory
Zargooshi 2001	Iran	Single-centre retrospective questionnaire survey	300 LKDs, 100 controls	Median 61 months (range 6-132)	QoL	SF-36
Zheng et al. 2014	China	Single-centre retrospective questionnaire survey	110 LKDs, 124 recipients	Median 39 months (range 1-106)	QoL, donation experience, Anxiety, Depression	SF-36, SAS, SDS, Standardized self-developed questions
Zorgdrager et al. 2019	Netherlands	Single-centre retrospective questionnaire survey	333 living kidney donors	Median 19 months (IQR 10-33)	Post-donation pain and movement disabilities	CCS and VAS

Abbreviations of terms: HALDN = Hand-assisted laparoscopic donor nephrectomy; HRQoL = Health-Related Quality of Life; IQR = interquartile range; LDN = Laparoscopic Donor Nephrectomy; LKDs = Living Kidney Donors; MIDN = Mini-Incision open Donor Nephrectomy; LOD = Living Organ Donation; NDD = NonDirected Donor; N/A = Not applicable; NKR = National Kidney Register; ODN = Open Donor Nephrectomy; QoL = Quality of Life; RCT = Randomized Controlled Trial; RPDN = retroperitoneoscopic donor nephrectomy; SD = Standard Deviation; VAS = Visual Analogue Scale

Abbreviations of instruments: AA = Anterior Approach Donor Nephrectomy; ACSA = Anamnestic Comparative Self-Assessment; BSI = Brief Symptoms Inventory; BAI = Beck Anxiety Inventory; BDI = Beck Depression Inventory; CCS = Carolinas Comfort Scale; CERQ = Cognitive Emotion Regulation Scale; CIS = Checklist Individual Strength-Fatigue Short Version; CSQ-8 = Client Satisfaction Questionnaire-8; DASS = Depression Anxiety Stress Scale; D-MHC-SF = Dutch Mental Health Continuum-Short Form; ELSA = English Longitudinal Study of Ageing Self-anchoring Scale; EPQ-RA = Eysenck Personality Questionnaire-Revised-Abbreviated; EQ-5D = EuroQol 5D; ESS = EULID (European Living Donation and Public Health Project) Satisfaction Survey; FSI = Fatigue Symptom Inventory ; GAD-2 = Generalized Anxiety Disorder-2 item scale; GHQ-12 = General health questionnaire-12; HADS = Hospital Anxiety and Depression Scale; HAM-A = Hamilton Anxiety Rating Scale; HAM-D = Hamilton Depression Rating Scale; HARP = Hand-Assisted Retroperitoneoscopic donor nephrectomy; LDEQ = Living Donor Expectations Questionnaire; LOT-R = Life Orientation Test-Revised; MFI-20 = Multidimensional

Fatigue Inventory-20; MINI-plus = Mini International Neuropsychiatric Interview; PANAS = Positive and Negative Affect Scale; PHQ = Patient Health Questionniare; PSS-10 = Perceived Stress Scale; SAS = Zung Self-rating Anxiety Scale; SCL-90 = Symptom Checklist-90; SDS = Zung Selfrating Depression Scale; SF-36 = Short Form-36 Health Survey; SOCS = Sense of Coherence Scale; SSL = Social Support List-Interactions and Discrepancies; SSQ = Social Support Questionnaire; SSRS = Social Support Rating Scale for Chinese; STAI = State-Trait Anxiety Inventory; SWLS = Satisfaction with life scale; WHOQOL-BREF= World Health Organization quality of life brief questionnaire

* Multiple articles from the same dataset/study
